# *AoPEX1* and *AoPEX6* Are Required for Mycelial Growth, Conidiation, Stress Response, Fatty Acid Utilization, and Trap Formation in *Arthrobotrys oligospora*

**DOI:** 10.1128/spectrum.00275-22

**Published:** 2022-03-24

**Authors:** Qianqian Liu, Dongni Li, Kexin Jiang, Ke-Qin Zhang, Jinkui Yang

**Affiliations:** a State Key Laboratory for Conservation and Utilization of Bio-Resources, Key Laboratory for Microbial Resources of the Ministry of Education, School of Life Sciences, Yunnan University, Kunming, People’s Republic of China; University of Molise

**Keywords:** *Arthrobotrys oligospora*, peroxisome biogenesis-genes, conidiation, fatty acid utilization, trap formation, transcriptome analysis

## Abstract

*Arthrobotrys oligospora* (*A. oligospora*) is a typical nematode-trapping (NT) fungus that can capture nematodes by producing adhesive networks. Peroxisomes are single membrane-bound organelles that perform multiple physiological functions in filamentous fungi. Peroxisome biogenesis proteins are encoded by *PEX* genes, and the functions of *PEX* genes in *A. oligospora* and other NT fungi remain largely unknown. Here, our results demonstrated that two *PEX* genes (*AoPEX1* and *AoPEX6*) are essential for mycelial growth, conidiation, fatty acid utilization, stress tolerance, and pathogenicity in *A. oligospora*. *AoPEX1* and *AoPEX6* knockout resulted in a failure to produce traps, conidia, peroxisomes, and Woronin bodies and damaged cell walls, reduced autophagosome levels, and increased lipid droplet size. Transcriptome data analysis showed that *AoPEX1* and *AoPEX6* deletion resulted in the upregulation of the proteasome, membranes, ribosomes, DNA replication, and cell cycle functions, and the downregulation of MAPK signaling and nitrogen metabolism. In summary, our results provide novel insights into the functions of *PEX* genes in the growth, development, and pathogenicity of *A. oligospora* and contribute to the elucidation of the regulatory mechanism of peroxisomes in trap formation and lifestyle switching in NT fungi.

**IMPORTANCE** Nematode-trapping (NT) fungi are important resources for the biological control of plant-parasitic nematodes. They are widely distributed in various ecological environments and capture nematodes by producing unique predatory organs (traps). However, the molecular mechanisms of trap formation and lifestyle switching in NT fungi are still unclear. Here, we provided experimental evidence that the *AoPEX1* and *AoPEX6* genes could regulate mycelial growth and development, trap formation, and nematode predation of *A. oligospora*. We further analyzed the global transcription level changes of wild-type and mutant strains using RNA-seq. This study highlights the important role of peroxisome biogenesis genes in vegetative growth, conidiation, trap formation, and pathogenicity, which contribute to probing the mechanism of organelle development and trap formation of NT fungi and lays a foundation for developing high-efficiency nematode biocontrol agents.

## INTRODUCTION

Peroxisomes are conserved organelles in almost all eukaryotic cells that are rich in enzymes that participate in various biochemical metabolic processes, such as fatty acid β-oxidation, glyoxylate detoxification, cholesterol synthesis, regulation of reactive oxygen species, assimilation of methanol in yeast, and biosynthesis of plasmalogen in mammals (1–3). In eukaryotes, peroxisomes are involved in various biochemical processes; for example, in yeast, the peroxisome is the only site for fatty acid β-oxidation, which is critical for cellular maintenance of fatty acid homeostasis (4, 5). In plants, peroxisomes are involved in embryonic development, photorespiration, host resistance, nitrogen and sulfur compound metabolism, plant hormone synthesis, and the glyoxylate cycle (6–9). Peroxisomes also play vital roles in filamentous fungi, as various fungal metabolites are partially synthesized within them. Peroxisome-deficient fungi fail to grow in minimal medium containing fatty acids as the sole carbon source and exhibit delayed growth and aberrant organelle morphologies (10–12).

Recent advances have resulted in the identification of over 30 *PEX* genes, which are essential factors for peroxisome biogenesis in various species from yeast to humans (13); these have been found to be involved in matrix protein import, membrane biogenesis, and organelle proliferation (3). Both *PEX1* and *PEX6* encode an AAA-type ATPase and are involved in importing peroxisomal matrix proteins by mediating the recycling of peroxisomal membrane targeting signals (PTS) receptors in Saccharomyces cerevisiae. Kimura et al. first reported that the *PEX6* gene is necessary for *Colletotrichum lagenarium*-mediated infection and reduces fatty acid utilization. In addition, the same authors reported that PST1-mediated matrix protein transport could not be performed normally in the *PEX6* mutant strain, indicating the involvement of peroxisomal metabolism in the pathogenicity of plant fungal pathogens (14). In Magnaporthe oryzae, *PEX1* is involved in regulating growth and sporulation ability, appressorium morphology, pathogenicity, and other biological processes (15, 16). Moreover, some other *PEX* genes have been identified to be involved in many functions. For example, loss of the *PEX19* gene affects the transport process of matrix and membrane proteins, resulting in a complete loss of pathogenicity (17). Knockout of the *PEX14/17* gene was shown to reduce fat utilization ability, reactive oxygen resistance, and pathogenicity (18). Taken together, *PEX* genes participate in the regulation of various biological processes in filamentous fungi, including pathogenicity, mycelial growth, and fat utilization, sporulation, and reactive oxygen degradation capacities.

Nematode-trapping (NT) fungi are broadly distributed in terrestrial and aquatic ecosystems. They can trap and digest nematodes using specialized trapping devices (traps), such as adhesive networks and knobs and constricting rings. The development of these traps is a key indicator of their switch from saprophytic to predacious lifestyles (19–21). *Arthrobotrys oligospora* is a representative species of NT fungi that produces adhesive networks to capture nematodes (21). To date, the whole-genome sequence of *A. oligospora* has been reported and annotated, and the trap formation pathway has also been proposed through proteomics analysis (21). The ultrastructure of *A. oligospora* has been observed through transmission electron microscopy, revealing that the structure of trap cells significantly differs from that of vegetative hyphae cells. There are many ultrastructural bodies in the hyphal cells of traps, known as electron-dense (ED) bodies (22). Since these EDs have catalase and d-amino acid oxidase activities, they may belong to peroxisomes based on their biochemical properties (23). In addition, Woronin bodies, organelles derived from peroxisomes, play an important role in various biological processes such as conidia development, infective structure formation, stress resistance, and adaptation to nutrient-deficient environments (24). In summary, peroxisomes are closely related to trap formation. However, the role of peroxisomes in trap formation in NT fungi remains largely unknown.

To investigate the biological functions and regulatory mechanisms of peroxisomes in NT fungi, we characterized *A. oligospora PEX1* and *PEX6* homologs (*AoPEX1* and *AoPEX6*, respectively) through gene knockout, and related phenotypic experiments were used to infer the roles of *AoPEX1* and *AoPEX6* in mycelial development and nematode predation. Moreover, transcriptome technology was used to analyze global changes in gene transcription levels between the wild-type (WT) strain and mutants, laying a foundation for further clarifying the regulatory mechanism of peroxisomal proteins in NT fungi trap formation.

## RESULTS

### Sequence and phylogenetic analyses of *AoPex1* and *AoPex6*.

Analysis using the Compute pI/*M*_w_ tool of the ExPASy database revealed that *AoPEX1* encodes a protein with 1,194 amino acid residues, a theoretical molecular mass of 127.88 kDa, and pI of 4.74, while *AoPEX6* encodes a protein containing 1,183 amino acids residue, a molecular mass of 126.95 kDa, and pI of 8.45. The functional domains of the two proteins were analyzed using the InterProScan database; both *AoPex1* and *AoPex6* contain an ATPase AAA core domain (IPR003959) and an AAA-type ATPase domain (IPR003593), as well as an ATPase AAA CS function site (IPR003960). Unexpectedly, *AoPex1* has an extra PEX1-N psi beta-barrel domain (IPR015432) and an AAA lid 3 domain (IPR041569), unlike *AoPex6*. Furthermore, *AoPex1* is highly similar to the orthologs of three other NT fungal species, Arthrobotrys flagrans (97.1%), Dactylellina haptotyla (88.5%), and Drechslerella brochopaga (84.9%). In contrast, it shares moderate similarity (41.1–46.1%) with orthologs from other filamentous fungi, such as Aspergillus nidulans, M. oryzae, and Neurospora crass. Similarly, the *AoPex6* sequence is identical to those of orthologs found in the NT fungi A. flagrans (97.2%), D. haptotyla (94.4%), and D. brochopaga (90.6%) and less so compared with other filamentous fungi (55.4–57.7%) or Saccharomyces cerevisiae (9.4%). Moreover, a phylogenetic tree of orthologous Pex proteins from various fungi was constructed. As expected, Pex1 or Pex6 orthologs from NT fungi clustered into two independent branches (Fig. S1 in the online supplementary material).

### *AoPEX1* and *AoPEX6* regulate mycelial growth and the number of cell nuclei.

**FIG 1:**
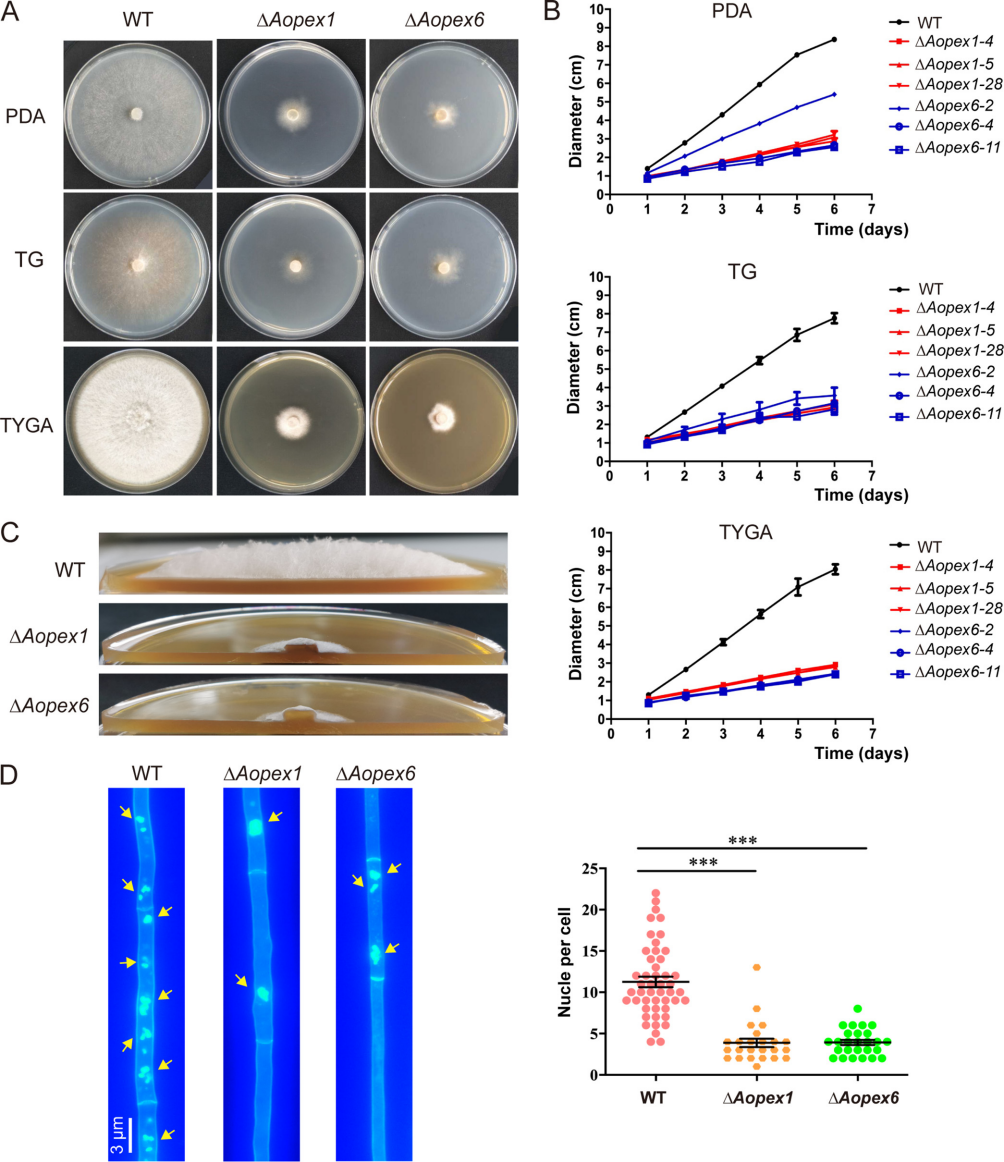
Comparison of hyphal growth of wild-type (WT) and mutant strains in *A. oligospora*. (A) Colonial shapes of fungal strains cultured on PDA, TG, and TYGA plates at 28°C. The strains were photographed after 6 days of incubation. (B) The quantification data for (A). (C) Aerial hyphae of the WT and mutant strains cultured on TYGA medium for 5 d. (D) Hyphae of the WT and each mutant strain were stained with CFW and 4′,6-diamidino-2-phenylindole (DAPI) after the fungal strains were grown for 7 days on CMY medium. One hundred hyphal cells were randomly selected for counting cell nuclei. Bar: 3 μm. Measurements represent the average of three independent experiments. The asterisk indicates a significant difference between the mutant and the WT strains (***P < 0.001).

The *AoPEX1* and *AoPEX6* genes were disrupted as described in Materials and Methods (Fig. S2 in the online supplementary material), and three mutants of each gene were randomly selected for subsequent analysis. The hyphae of the Δ*Aopex1* and Δ*Aopex6* mutant strains showed obvious growth defects compared with those of the WT strains (Fig. 1A), and their colony diameters were reduced by 65 and 62.5%, respectively (Fig. 1B). Subsequently, using a side-shot approach for the aerial hyphae, we observed that the Δ*Aopex1* and Δ*Aopex*6 mutants displayed sparse aerial hyphae, whereas the WT strain showed dense aerial hyphae following culturing on TYGA medium (Fig. 1C). Moreover, the number of nuclei in hyphal cells from the Δ*Aopex1* mutant (3–4 nuclei per cell) and Δ*Aopex6* mutant (4–5 nuclei per cell) was less than that in the WT strain (11–13 nuclei per cell) (Fig. 1D).

### Absence of the *AoPEX1* and *AoPEX6* genes eliminated conidiation, trap formation, and pathogenicity.

**FIG 2:**
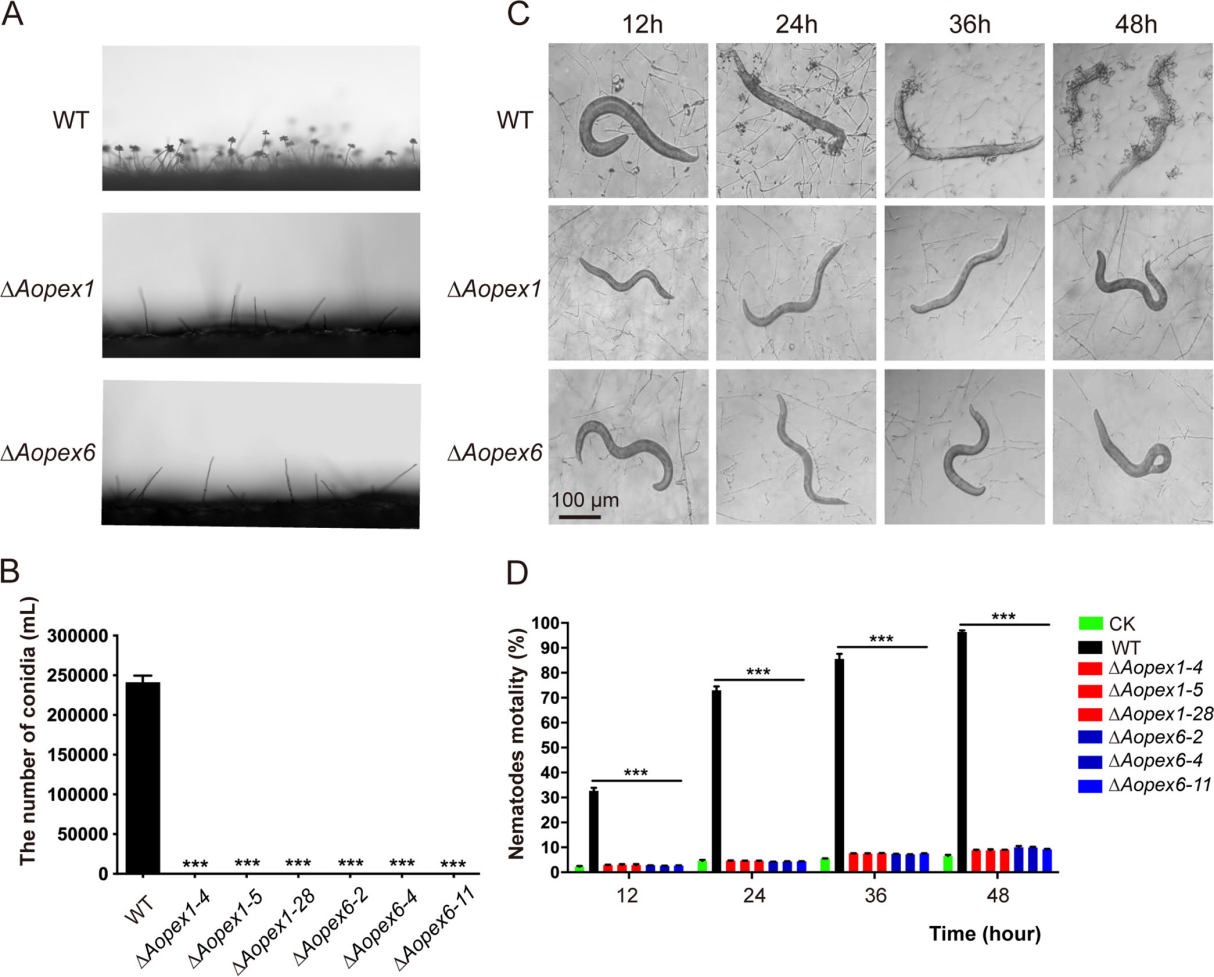
Comparison of sporulation, trap formation, and nematocidal activity in the wild-type (WT) and mutant strains. (A) Conidiophore differentiation in the WT and mutant lines. (B) Spore yields of the WT and mutant lines. (C) Trap formation in the WT and mutant lines induced by nematodes at different time points. Bar: 100 μm. (D) The percentage of nematode mortality by the WT and mutant lines at different time points. CK is the negative control using WA plate to assess the viability of the C. elegans in the absence of fungal strains. The asterisk (B and D) indicates a significant difference between the mutant and the WT strains (***P < 0.001).

Compared with the WT strain, the number and density of conidiophores in Δ*Aopex1* and Δ*Aopex6* mutant strains were remarkably decreased, and neither mutant could produce conidia on the conidiophore (Fig. 2A–B), indicating that the mutants lost the ability to produce conidia. Transcriptome data analysis revealed the increased downregulation of sporulation-related genes in mutant strains compared with the WT strain (Fig. S3 in the online supplementary material). Trap formation was induced by adding nematodes (Caenorhabditis elegans) to the WA plates. Results showed several traps on the plates containing the WT strain, but none were observed on the plates containing *ΔAopex1* and *ΔAopex6* after adding nematodes for 12, 24, 36, and 48 h (Fig. 2C). Moreover, the nematode mortality rate was remarkably decreased in Δ*Aopex1* and Δ*Aopex6* relative to WT (Fig. 2D). Taken together, these results indicate that deleting *AoPEX1* and *AoPEX6* genes led to complete elimination of sporulation and trap formation and a remarkable decrease in the ability to trap nematodes in A. oligospora.

### *AoPEX1* and *AoPEX6* are critical to stress response.

**FIG 3:**
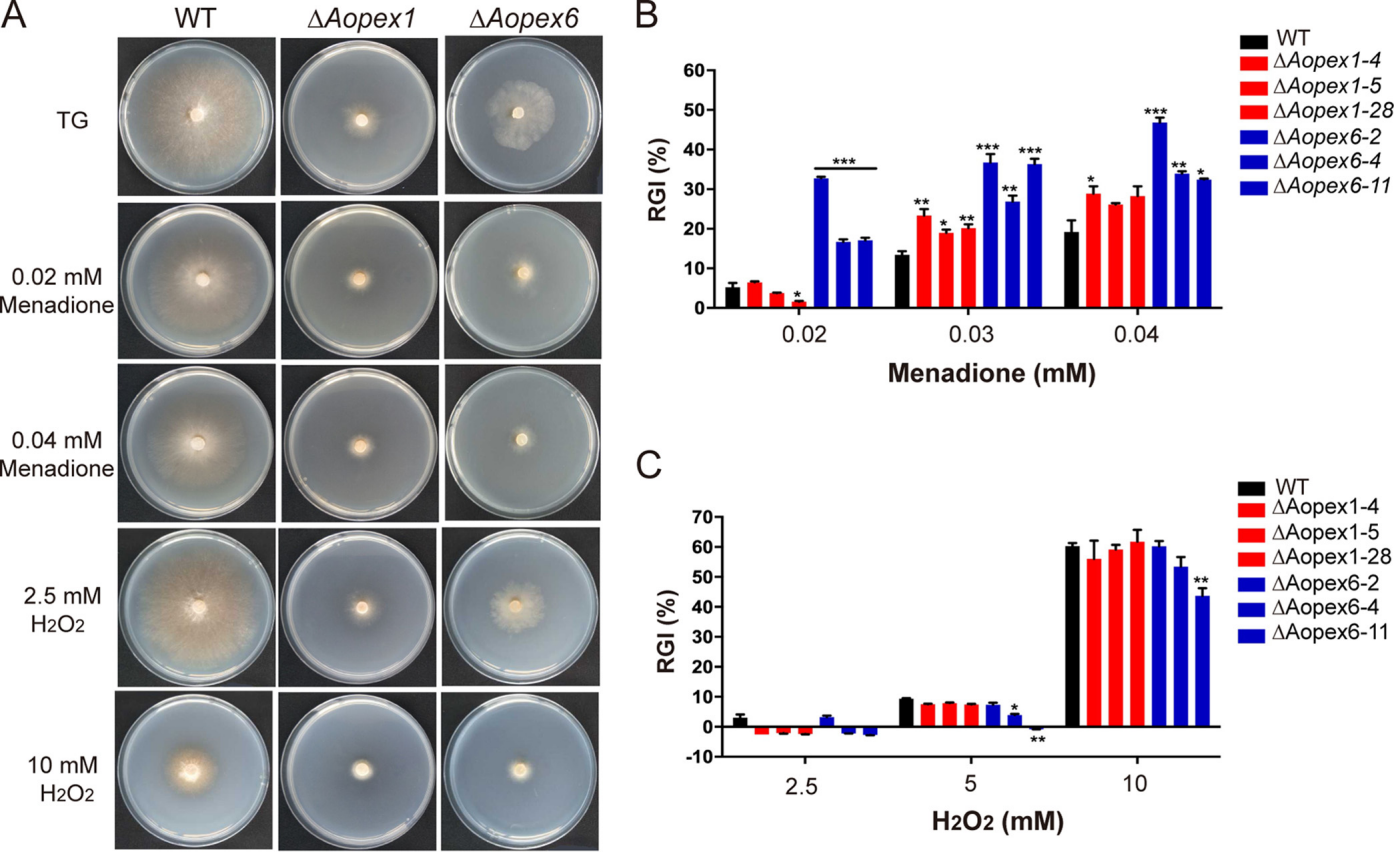
Comparison of stress tolerance to oxidative agents between wild-type (WT) and mutant strains of *A. oligospora*. (A) Colony morphologies of the WT and mutant strains incubated on TG medium supplemented with menadione or H_2_O_2_. (B) Relative growth inhibition (RGI) values of the WT and mutants after incubation on TG medium supplemented with 0.02–0.04 mM menadione for 6 days. (C) RGI values for the WT and mutant strains after incubation on TG medium supplemented with 2.5–10 mM H_2_O_2_ for 6 days. The asterisk (B and C) indicates a significant difference between the mutant and the WT strains (*P < 0.05, **P < 0.01, ***P < 0.001).

To investigate the role of *AoPEX1* and *AoPEX6* under different chemical stress conditions, the mycelial growth of the WT and mutant strains was analyzed by comparing the relative growth inhibition (RGI) values on TG medium supplemented with various chemical agents. In osmotic sensitivity tests, Δ*Aopex1* mutants had reduced RGI values under low concentrations of NaCl and sorbitol stress conditions, while the Δ*Aopex6* mutant displayed greater sensitivity to 0.1 M NaCl, with considerably lower RGI values compared with WT (Fig. S4A in the online supplementary material). However, when growing on a medium containing 0.75 M sorbitol and 0.3 M NaCl, the sensitivity of the mutant strains was reduced (Fig. S4B and C). Next, we tested the oxidative stress response by adding menadione and H_2_O_2_ at different concentrations to the medium (Fig. 3A). The results showed that both Δ*Aopex1* and Δ*Aopex*6 were more sensitive to menadione, and their RGI values were substantially increased compared with WT (Fig. 3B). However, the growth of the mutant strains was not inhibited by H_2_O_2_ treatment (Fig. 3C).

To investigate whether *AoPEX1* or *AoPEX6* plays a role in cell wall biosynthesis in A. oligospora, we examined how the mutants responded to a range of cell wall stress agents (Fig. S5A in the online supplementary material). The colony diameters of the mutants were remarkably smaller than those of the WT strain when cultured on medium supplemented with 0.05 mg/mL Congo red (CR) (Fig. S5B). There was no significant difference in the RGI values between mutant and WT strains after adding SDS (Fig. S5C). Therefore, deleting *AoPEX1* and *AoPEX6* results in defective cell wall biosynthesis in A. oligospora.

### *AoPEX1* and *AoPEX6* play an important role in fatty acid utilization and hyphal morphology.

**FIG 4:**
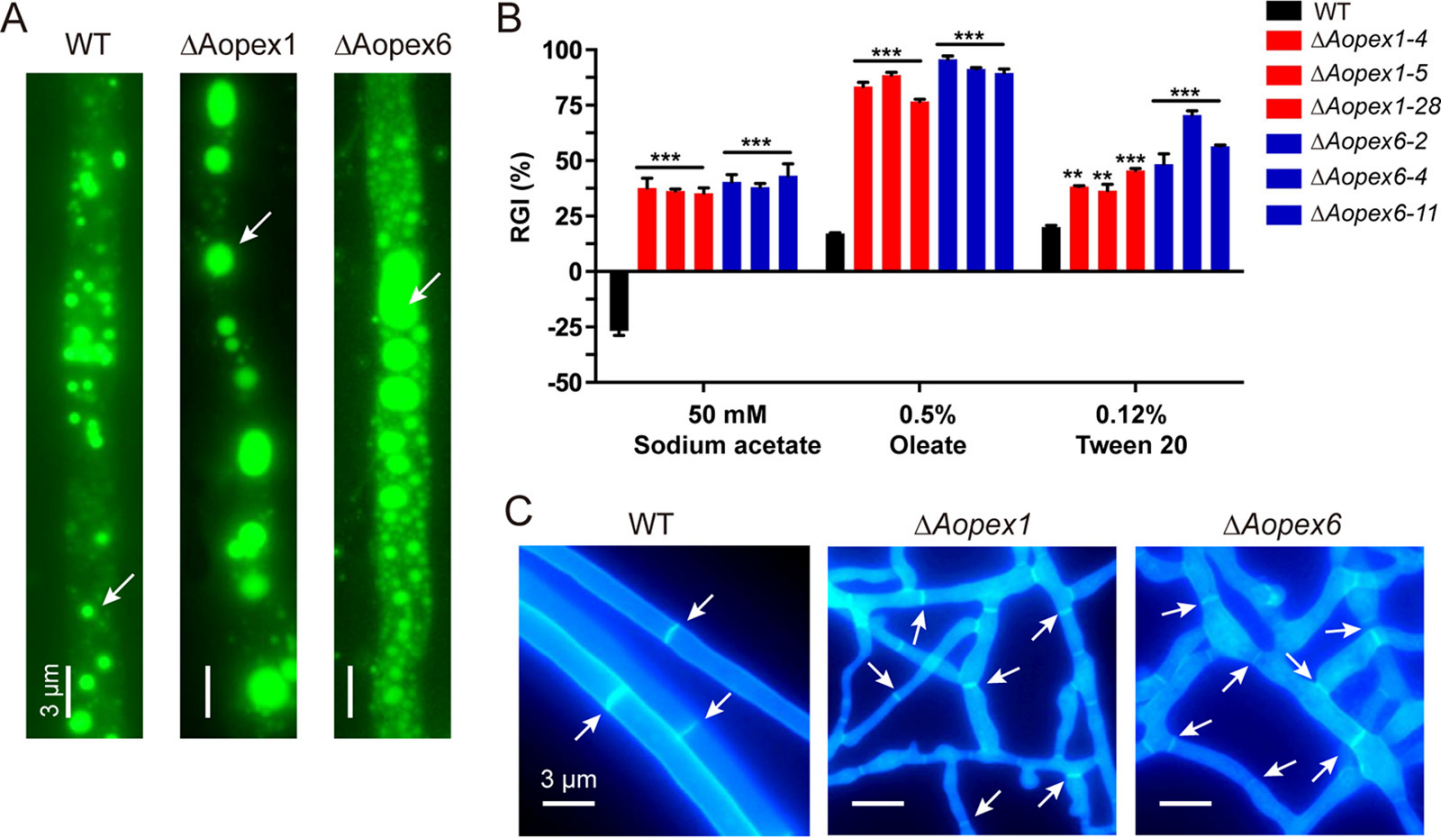
Comparison of fatty acid utilization and hyphae morphology between wild-type (WT) and mutant strains of *A. oligospora*. (A) The LDs of WT, Δ*Aopex1*, and Δ*Aopex6* strains were stained with BODIPY. Bar: 3 μm. (B) RGI values of fungal strains under different fatty acids as the only carbon source. The asterisk indicates a significant difference between the mutant and the WT strains (**P < 0.01, ***P < 0.001). (C) The CFW staining of WT and mutant strains. Bar: 3 μm.

Boron dipyrromethene dyes (BODIPY) specifically stain lipid droplets (LDs) composed of neutral lipids (25). Following hyphal staining and imaging, we found that the LD volumes of Δ*Aopex1* and Δ*Aopex6* were significantly enlarged compared with those of WT (Fig. 4A). Furthermore, to clarify the effect of *AoPEX1* and *AoPEX6* on fatty acid utilization, the vegetative growth of the WT and mutant strains on MM medium containing different fatty acids, including sodium acetate, oleate, and Tween 20 as the sole carbon source, was assessed. medium for 6 days, the RGI values of Δ*Aopex1* and Δ*Aopex6* mutants were significantly increased After incubation in fatty acid as the sole carbon, with oleic acid being the most significant. For example, the RGI value of the Δ*Aopex1* and Δ*Aopex6* mutants in 0.5% oleate increased by 79 and 81%, respectively, relative to that of the WT strain (Fig. 4B). These results indicate that *AoPEX1* and *AoPEX6* are required for fatty acid utilization in A. oligospora.

Subsequently, hyphae cultured on a CMY plate for 7 days were observed by staining with calcofluor white (CFW) dye. The results showed that Δ*Aopex1* and Δ*Aopex6* hyphae were enlarged and deformed and formed more branches to connect to the network structure compared with the WT strain (Fig. 4C).

### *AoPEX1* and *AoPEX6* regulate autophagy and biogenesis of peroxisomes and Woronin bodies.

**FIG 5:**
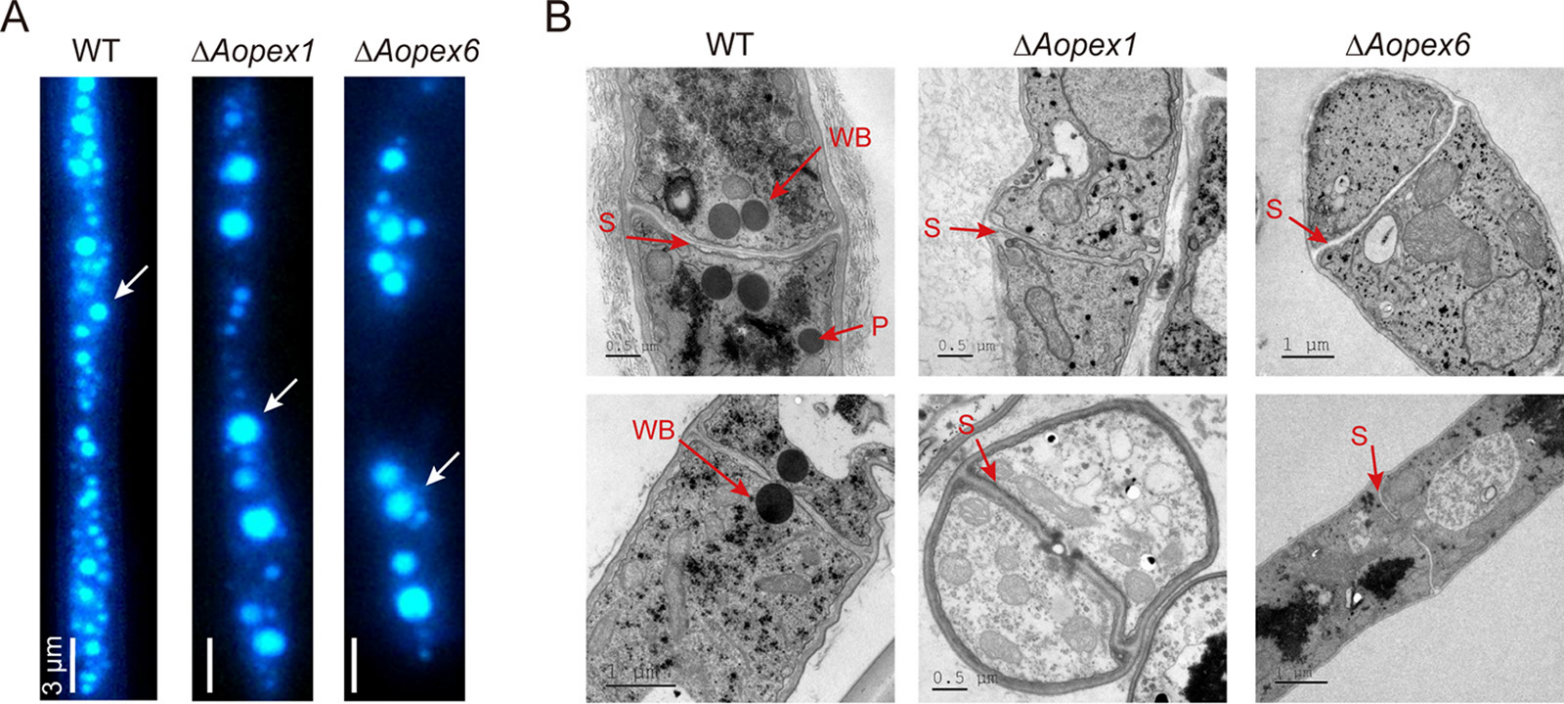
Observation of autophagosomes and ultrastructure of peroxisomes and Woronin bodies in the wild-type (WT) and mutant strains. (A) The autophagosomes of WT strain, Δ*Aopex1*, and Δ*Aopex6* mutant strains were stained with MDC. Bar: 3 μm. (B) The WT and mutant strains were observed by transmission electron microscopy. WB: Woronin body; P: peroxisome; S: septum.

By staining with monodansylcadaverine (MDC) dye, we found that the number of autophagosomes in Δ*Aopex1* and Δ*Aopex6* mutants was decreased, but the volume of autophagosomes remarkably increased (Fig. 5A). In addition, transmission electron microscopy (TEM) results showed that the WT strain had Woronin bodies near the hyphal septum, and peroxisomes could be observed in the hyphae. However, Woronin bodies and peroxisomes were not observed in the Δ*Aopex1* and Δ*Aopex6* strains (Fig. 5B).

### Transcriptomic profile analysis of the WT and mutant strains.

To further explore the regulatory mechanisms of *AoPEX1* and *AoPEX6* in *A. oligospora*, we compared the transcriptomic profiles of the WT and mutant strains using RNA-seq technology. The mycelial samples of WT and mutant strains were collected and cultured in PD broth for 3 and 5 days, respectively, after which a cDNA library was constructed and sequenced for each sample. As a result, 41.6–53.7 million reads were obtained for each sample (Table S1 in the online supplemental material). The percentage of Phred-like quality scores at the Q30 level ranged from 92.5 to 94.0%, and the GC content ranged from 47.4 to 48.4% (Table S2). Principal-component analysis showed that the WT and mutant strains were located in different quadrants at various time points, indicating that their transcription profiles significantly differed and higher similarity appeared in the three replicates of the samples (Fig. S6). Meanwhile, 15 genes associated with the nitrogen compound metabolic process, nitrogen metabolism, and MAPK signaling were selected to verify the transcriptomic data by reverse transcription-quantitative PCR (RT-qPCR) (Table S3). The transcriptome showed downregulation of nitrogen compound metabolic processes, nitrogen metabolism, and MAPK signaling-related gene levels. In addition, RT-qPCR verified that mRNA levels were also downregulated at 3 and 5 days—for WT and mutant strains, respectively—indicating a relatively high consistency between the RNA-seq and RT-qPCR analyses (Fig. S7).

**FIG 6:**
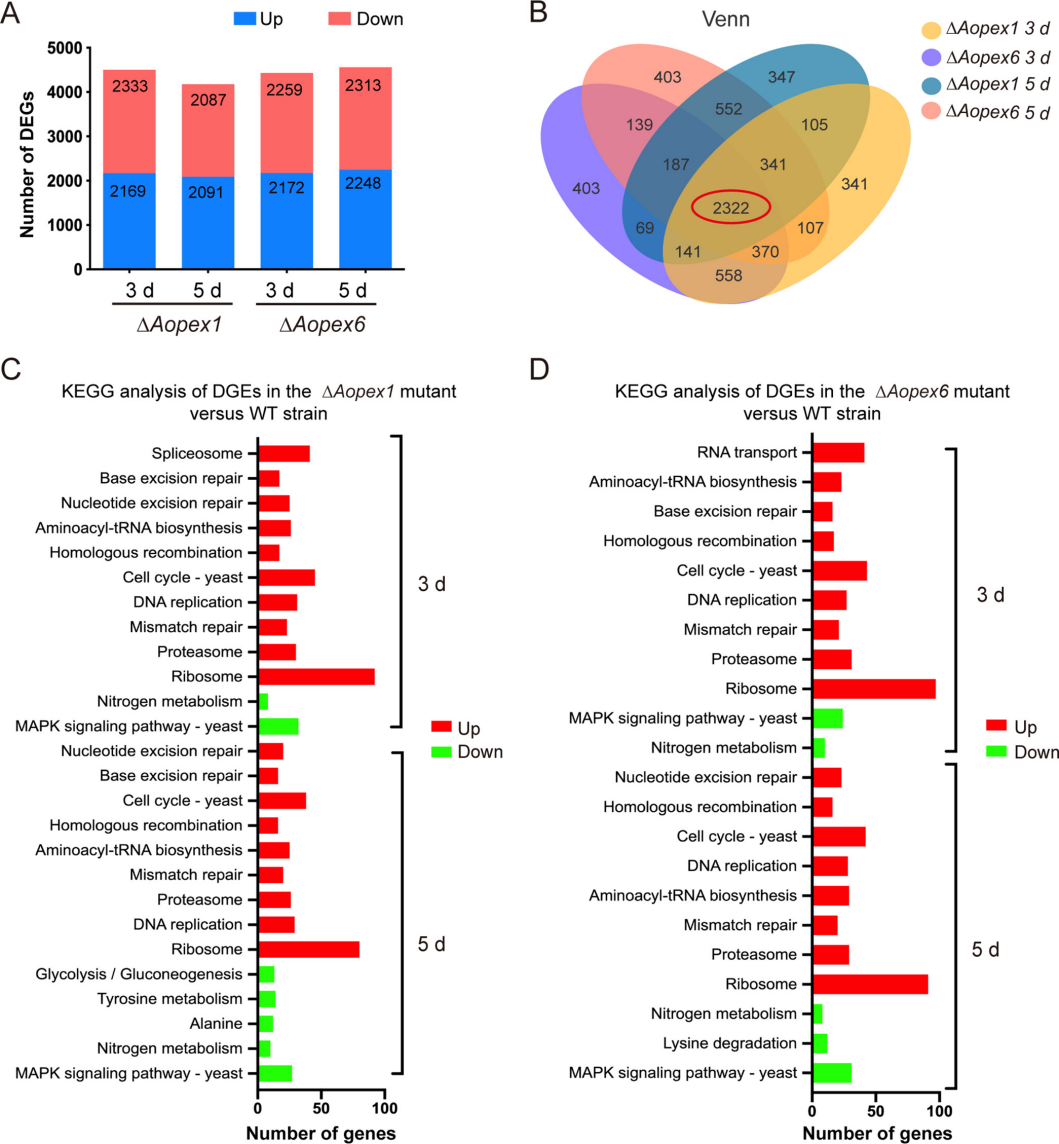
Transcription analysis in wild-type (WT), Δ*Aopex1*, and Δ*Aopex6* mutant strains. (A) DEGs on the 3rd and 5th days. Red, upregulated DEGs; green, downregulated DEGs. (B) Venn analysis of WT, Δ*Aopex1*, and Δ*Aopex6* mutant strains at two time points. (C) KEGG enrichment analysis of DEGs in WT and Δ*Aopex1* strains. (D) KEGG enrichment analysis of DEGs in WT and Δ*Aopex6* strains. DEGs, differentially expressed genes.

The Δ*Aopex1* and Δ*Aopex6* strains were found to have 4,496 and 4,178 and 4,431 and 4,560 differentially expressed genes (DEGs) at 3 and 5 days, respectively (Fig. 6A). Between the WT and mutant strains, there were 4285 and 4064 and 4421 and 4189 co-expressed genes at 3 and 5 d, respectively (Fig. S8 in the online supplemental material). Afterward, 2,322 contigs shared between the Δ*Aopex1* and Δ*Aopex6* mutants were found at all time points through Venn analysis (Fig. 6B). Gene Ontology (GO) enrichment analysis was performed to analyze the functional categories of these DEGs. On the 3rd day, the most enriched GO terms in the Δ*Aopex1* strain were associated with molecular functions (MF), including aminoacyl-tRNA ligase activity and ligase activity; seven cellular components (CC), such as proteasome storage granule, endopeptidase complex, proteasome complex, and protein folding; and 11 biological processes (BP) such as tRNA aminoacylation for protein translation, protein catabolic process, and DNA replication. The most downregulated processes were transcriptional regulatory activity, nitrogen metabolism, and primary metabolism. On the 5th day, the upregulated GO terms included the cell cycle, cytoplasmic part, and ribosomal subunits, while the downregulated GO term was RNA metabolism (Fig. S9A). In addition, the significantly upregulated and downregulated GO terms were very similar between the Δ*Aopex6* and Δ*Aopex1* strains at 3 and 5 days (Fig. S9B).

Furthermore, pathway enrichment in the WT and Δ*Aopex1/*Δ*Aopex6* strains was compared and analyzed using the Kyoto Encyclopedia of Genes and Genomes (KEGG) analysis. Compared with the WT, there were 10 and 9 significantly upregulated pathways and 2 and 5 downregulated pathways in the Δ*Aopex1* mutant at 3 and 5 days, respectively. However, the Δ*Aopex6* mutant had 10 and 8 significantly upregulated pathways and 2 and 3 significantly downregulated pathways. On the thirrd day, the same significantly upregulated pathways, including cell cycle-yeast, ribosome, and proteasome, were enriched in both Δ*Aopex1* and Δ*Aopex6* mutants. In addition, the KEGG enrichment of DEGs on the third and fifth days was very similar, and the difference was that glycolysis/gluconeogenesis and tyrosine metabolism were additionally enriched in the Δ*Aopex1* mutant, and lysine degradation was additionally enriched in the Δ*Aopex6* mutant (Fig. 6C and D).

Based on GO and KEGG analyses, we analyzed the expression of several genes related to catalase expression. The results showed that most genes related to the oxidative stress response were altered in mutants compared with those in the WT at 3 and 5 days, including *cat* (AOL_s00188g243), *cat-2* (AOL_s00006g411 and AOL_s00076g488), *nox-1* (AOL_s00193g69), *nox-2* (AOL_s00007g557), *noxR* (AOL_s00054g538), and *sod-2* (AOL_s00170g93). The expression levels of *nox-2*, *noxR*, and *sod-2* were considerably downregulated in Δ*Aopex1* and Δ*Aopex6* mutants at 3 and 5 days, whereas that of *cat* was upregulated (Table 1). In addition, we also analyzed the expression of genes related to cell wall biosynthesis, and the results showed that except for *hex* (AOL_s00112g89), other genes were significantly altered in the mutants at 3 and 5 days, including *glu* (AOL_s00083g375), *chs-3* (AOL_s00078g76), *chs* (AOL_s00075g119), *trs* (AOL_s00097g268), and *gls* (AOL_s00054g491). Furthermore, *chs*, *chs-3*, *trs*, and *gls* transcripts were conspicuously downregulated in the mutants, whereas that of *glu* was considerably upregulated in the mutants (Table 1).

**TABLE 1:** Transcriptional response to *Aopex1* and *Aopex6* deletion by the genes involved in oxidation stress response and cell wall biosynthesis in comparative transcriptome analysis^*a*^

Locus	Function annotation	Expressional levels					
		TPM-3 d			TPM-5 d		
		WT	Δ*Aopex1*	Δ*Aopex6*	WT	Δ*Aopex1*	Δ*Aopex6*
Genes involved in oxidation stress response							
AOL_s00173g374	*cat*, catalase	114.08	108.57	157.15	221.52	271.79	207.81
AOL_s00188g243	*cat*, catalase	0.05	0.34	0.2	0	0.37	0.06
AOL_s00006g411	*cat2*, catalase	362.47	182.99	337.40	112.35	241.7	272.67
AOL_s00076g488	*cat2*, catalase	2.42	2.06	1.09	4.85	11.82	3.82
AOL_s00193g69	*nox-1*, NADPH oxidase	116.94	55.24	54.31	112.35	119.31	87.49
AOL_s00007g557	*nox-2*, NADPH oxidase	150.80	26.48	29.05	119.68	26.63	37.86
AOL_s00054g538	*noxR*, NADPH oxidase regulator	8.83	4.17	4.52	9.19	6.85	3.00
AOL_s00007g292	*sod*, superoxide dismutase	57.92	46.19	50.59	31.22	50.78	55.37
AOL_s00054g687	*sodB*, superoxide dismutase	327.69	278.85	267.48	302.02	432.79	344.10
AOL_s00170g93	*sod-2*, superoxide dismutase	19.72	2.21	4.63	20.50	11.63	4.52
Genes involved in cell wall biosynthesis							
AOL_s00078g76	*chs-3*, chitin synthases	179.05	41.50	57.1	274.17	76.86	44.69
AOL_s00112g89	*hex*, hexokinase	673.25	324.68	331.42	249.00	175.31	251.03
AOL_s00075g119	*chi*, chitin synthase	122.65	36.49	45.59	217.50	39.69	20.01
AOL_s00097g268	*trs*, trehalose synthase	375.64	68.82	73.41	401.90	47.43	64.96
AOL_s00083g375	*glu*, beta-glucosidase	3.54	15.63	12.96	3.63	10.13	8.76
AOL_s00054g491	*gls*, 1,3-beta-glucan synthase	363.28	75.11	127.99	657.36	140.19	113.51

a TPM, transcripts per kilobase million. WT, wild-type strain; Δ*Aopex1*, *Aopex1* deletion mutant; Δ*Aopex6*, *Aopex6* deletion mutant; -3 d and -5 d, samples of the WT, Δ*Aopex1* and Δ*Aopex6* mutant strains in vegetative growth stage. Locus numbers and function were annotated according to the A. oligospora genome assembly (https://www.ncbi.nlm.nih.gov/).

### Cluster analysis of transcriptome.

**FIG 7:**
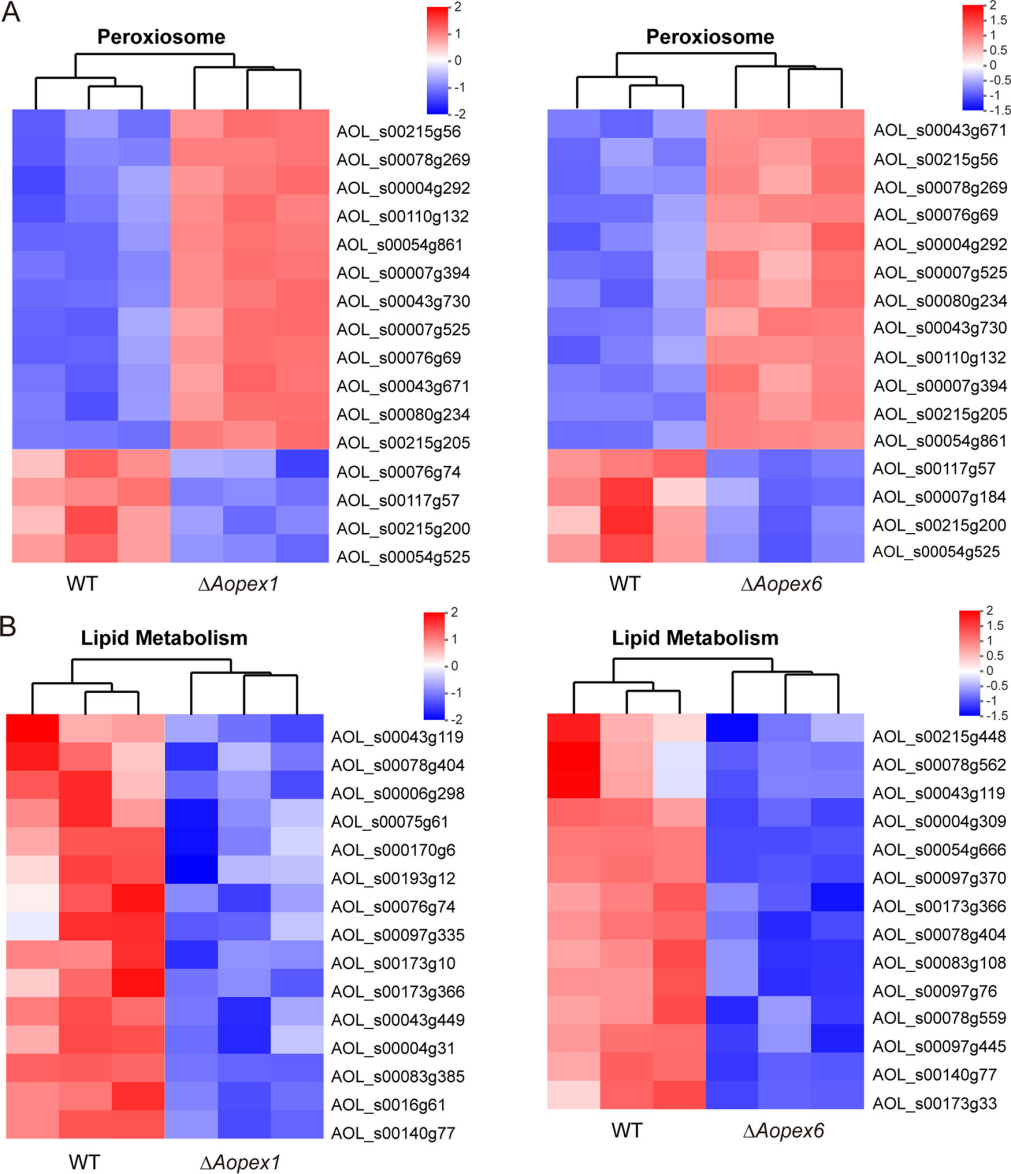
Differentially expressed genes (DEGs) associated with peroxisome and lipid metabolism. (A) Heat map showing the DEGs involved in peroxisomes. (B) Heat map showing the DEGs involved in lipid metabolism. Gene expression patterns are in log_10_-scale. Red boxes, upregulated clusters; blue boxes, downregulated clusters.

Based on GO and KEGG analyses, we performed a cluster analysis of genes related to peroxisome and lipid metabolism. The results showed that the up- and downregulated genes enriched in the Δ*Aopex1* and Δ*Aopex6* mutants were highly similar (Fig. 7A). Among the upregulated genes related to peroxisomes in Δ*Aopex1* and Δ*Aopex6* mutants, five genes were involved in peroxisome biosynthesis [Pex4 (AOL_s00078g269), Pex5 (AOL_s00043g671), Pex10 (AOL_s00054g861), Pex14 (AOL_s00004g292), and peroxisomal membrane protein Pmp47 (AOL_s00215g205)], three genes were involved in lipid fatty acid degradation [enoyl-CoA hydratase (AOL_s00215g56 and AOL_s00043g730) and long-chain acyl-CoA synthetase (AOL_s00007g525)], and two genes (AOL_s00110g132 and AOL_s00076g69) were related to the oxidative stress response. Three genes associated with peroxisomes were downregulated in Δ*Aopex1* and Δ*Aopex6* mutants, including sarcosine oxidase (AOL_s00117g57), long-chain acyl-CoA synthetase (AOL_s00215g200), and Pex13 (AOL_s00054g525) (Fig. 7A). Interestingly, the lipid metabolism-related genes were all less expressed in the mutant strains than in the WT strain. The Δ*Aopex1* and Δ*Aopex6* mutants shared four common genes, including AOL_s00043g119, AOL_s00078g404, AOL_s00173g366, and AOL_s00140g77 (Fig. 7B). Moreover, except for one gene (AOL_s00140g79) in Δ*Aopex6*, the other five genes involved in autophagy appeared in Δ*Aopex1*, and the expression levels of these genes in the mutant strains were lower than those in the WT (Fig. S10 in the online supplemental material).

## DISCUSSION

Peroxisomal biogenesis has been shown to be related to the pathogenicity of plant pathogenic fungi, such as *C. lagenarium* (14, 16, 26), Fusarium graminearum (14, 16, 26) and M. oryzae (14, 16, 26). There are 12 peroxin genes, including *PEX1*-*3*, *PEX5*, *PEX6*, *PEX10*, *PEX12*-*14*, *PEX16*, *PEX19*, and *PEX26*, which are all involved in the protein import machinery (27). Among them, Pex1 and Pex6, members of a large family of ATPases associated with diverse cellular activities, are the only proteins with ATP binding/hydrolysis domains involved in introducing peroxisome matrix proteins (28). In the present study, we identified *PEX1* and *PEX6* orthologs in *A. oligospora* (*AoPEX1* and *AoPEX6*, respectively), and phenotypic experiments showed that both orthologs played important roles in mycelial growth and development, stress response, and nematode predation and were essential for conidiation, trap formation, fatty acid utilization, and peroxisome and Woronin body biogenesis.

Previous studies have shown that knockout of the PEX genes *MoPEX1*, *MoPEX6*, and *AaPEX6* inhibited colony growth and spore production in M. oryzae (15, 16, 29) and Alternaria alternata, respectively (30). In the current study, the colony growth of Δ*Aopex1* and Δ*Aopex6* mutant strains was remarkably smaller than that of the WT, they did not produce aerial hyphae, and their colonies became compact and flat. In addition, CFW staining showed that Δ*Aopex1* and Δ*Aopex6* mutant strains exhibited hyphae enlargement compared with WT strains, and their hyphae branching became complicated, forming networks. Notably, in *A. oligospora*, loss of *AoPEX1* and *AoPEX6* leads to complete elimination of sporulation, differing from the marked reduction in conidiation caused by *AaPEX6* mutation in A. alternata (30). The eliminated sporulation capacity correlated with transcriptional repression of several sporulation-related genes in the Δ*Aopex1* and Δ*Aopex6* mutants (31). In addition, the number of nuclei in each hyphal cell was markedly decreased in Δ*Aopex1* and Δ*Aopex6* mutants than that in the WT strain. Taken together, our results revealed that both *AoPEX1* and *AoPEX6* are essential for *A. oligospora* mycelial growth and development.

In some plant-pathogenic fungi, loss of peroxisomes impairs pathogen growth because of inefficient utilization of the carbon sources from the host and the fungus itself (32). In M. oryzae, both *MoPEX1* and *MoPEX6* are involved in pathogenicity. Loss of *MoPEX1* or *MoPEX6* impaired vegetative growth, conidiation, and appressorium formation, and deletion of *MoPEX1* or *MoPEX6* resulted in the loss of peroxisomal integrity and failure to infect the plant host (15, 33). In addition, *PEX6* mutants lost pathogenicity in several plant-pathogenic fungi, including M. oryzae, *Colletotrichum gloeosporioides*, F. graminearum, and A. alternata due to defects in peroxisome biogenesis (14, 16, 30). Moreover, *PEX1* and *PEX6* deletion in M. oryzae led to a lack of Woronin bodies (13, 34), which are essential for efficient pathogenesis and survival during nitrogen starvation stress (35). In the present study, both Δ*Aopex1* and Δ*Aopex6* mutants lost peroxisomes and Woronin bodies. Furthermore, *AoPEX1* and *AoPEX6* knockout prevented trap formation and significantly decreased nematode infection. These findings showed that PEX1 and PEX6 orthologs are required for peroxisome and Woronin body biosynthesis, thus playing an indispensable role in the morphogenesis of infection structures and virulence of pathogenic fungi.

One of the most important functions of peroxisomes is fatty acid β-oxidation. Previous studies have found that deletion of *PEX5* and *PEX7* genes can also lead to the obstruction of fat utilization in M. oryzae (36). In F. graminearum, the absence of *PEX1* led to an increased accumulation of LDs and endogenous reactive oxygen species (37). In the present study, our results showed that the ability of Δ*Aopex1* and Δ*Aopex6* mutant strains to utilize sodium acetate, Tween 20, and oleic acid was significantly hindered. In addition, BODIPY staining revealed that LD volume in the two mutant strains increased, indicating that *AoPEX1* and *AoPEX6* knockout may inhibit fatty acid β-oxidation, resulting in larger LDs. Moreover, transcriptome data analysis revealed downregulated lipid metabolism, consistent with the results obtained from the phenotypic experiment. These results indicate that *PEX1* and *PEX6* affect the utilization of fatty acids in *A. oligospora* and possibly other fungi.

Peroxisomes contain various enzymes involved in the oxidative stress response, such as catalase, NADPH oxidase, and superoxide dismutase (38). Here, we found that deletion of *AoPEX1* and *AoPEX6* increases RGI values for several chemical stressors, including Congo red, NaCl, sorbitol, and menadione; however, mutant growth was not inhibited by treatment with SDS and H_2_O_2_. These phenotypic changes are consistent with the transcription of several genes involved in the oxidative stress response. Furthermore, biosynthesis of the cell wall was considerably downregulated in Δ*Aopex1* and Δ*Aopex6* mutants (38). These findings indicate that *PEX1* and *PEX6* orthologs are essential for stress response in A. oligospora.

Moreover, we observed that *AoPEX1* and *AoPEX6* are involved in regulating autophagy. Results showed that autophagosome number decreased in Δ*Aopex1* and Δ*Aopex6* mutants, whereas autophagosome volume markedly increased. By analyzing *AoPEX1* and *AoPEX6* transcriptome data, we found that the autophagy-related genes clustered into eight and six DEGs in the Δ*Aopex1* and Δ*Aopex6* mutants, respectively. Interestingly, except for one gene (AOL_s00140g79), the other five genes in Δ*Aopex6* were shared with Δ*Aopex1*, and their expression levels were downregulated in Δ*AoPEX1* and Δ*AoPEX6* mutants. Moreover, the deletion of autophagy-related genes has been observed to reduce trap formation in A. oligospora (24, 36). These results suggest that peroxisomes are closely related to autophagosome formation, thus may hinder trap formation in A. oligospora.

Numerous studies have shown that the interaction between fungi and their hosts can be studied using comparative transcriptome analysis (39). In the present study, we identified several DEGs between the WT and Δ*Aopex1* and Δ*Aopex6* mutant strains using RNA-seq. At the same time, GO and KEGG enrichment of DEGs were very similar between Δ*Aopex1* and Δ*Aopex6* mutants. The upregulated genes were mainly enriched in the ribosome, amino-tRNA biosynthesis, and proteasome, suggesting that protein synthesis is especially active during peroxisome biosynthesis. Furthermore, several DEGs were enriched in the cell cycle, spliceosome, and DNA replication and repair, suggesting that the absence of *Aopex1* and *Aopex6* may impair cell nucleus development. In fact, our results revealed that the number of cell nuclei was considerably decreased in Δ*Aopex1* and Δ*Aopex6* mutants. In addition, many downregulated genes were enriched in MAPK signaling, which is in line with previous reports (40). For example, deletion of G-protein signaling regulators caused a considerable reduction in conidiation and trap formation (41, 42), and disruption of MAPK signaling components, such as Slt2 and Fus3, resulted in trap formation defects in A. oligospora (40, 43, 44). A previous study hypothesized that the remains of organisms that died during mass extinctions are rich in carbon but poor in nitrogen, such that directly capturing nitrogen-rich living organisms would confer predatory fungi a competitive advantage over strictly saprophytic fungi (45). Recently, the nitrate assimilation pathway has been shown to be involved in trap formation by A. oligospora (46). Accordingly, in the present study, several DEGs were enriched in nitrogen metabolism and nitrogen compound metabolic processes. Therefore, our transcriptome analysis provides a good molecular basis for understanding phenotypic differences between WT A. oligospora and mutants.

**FIG 8:**
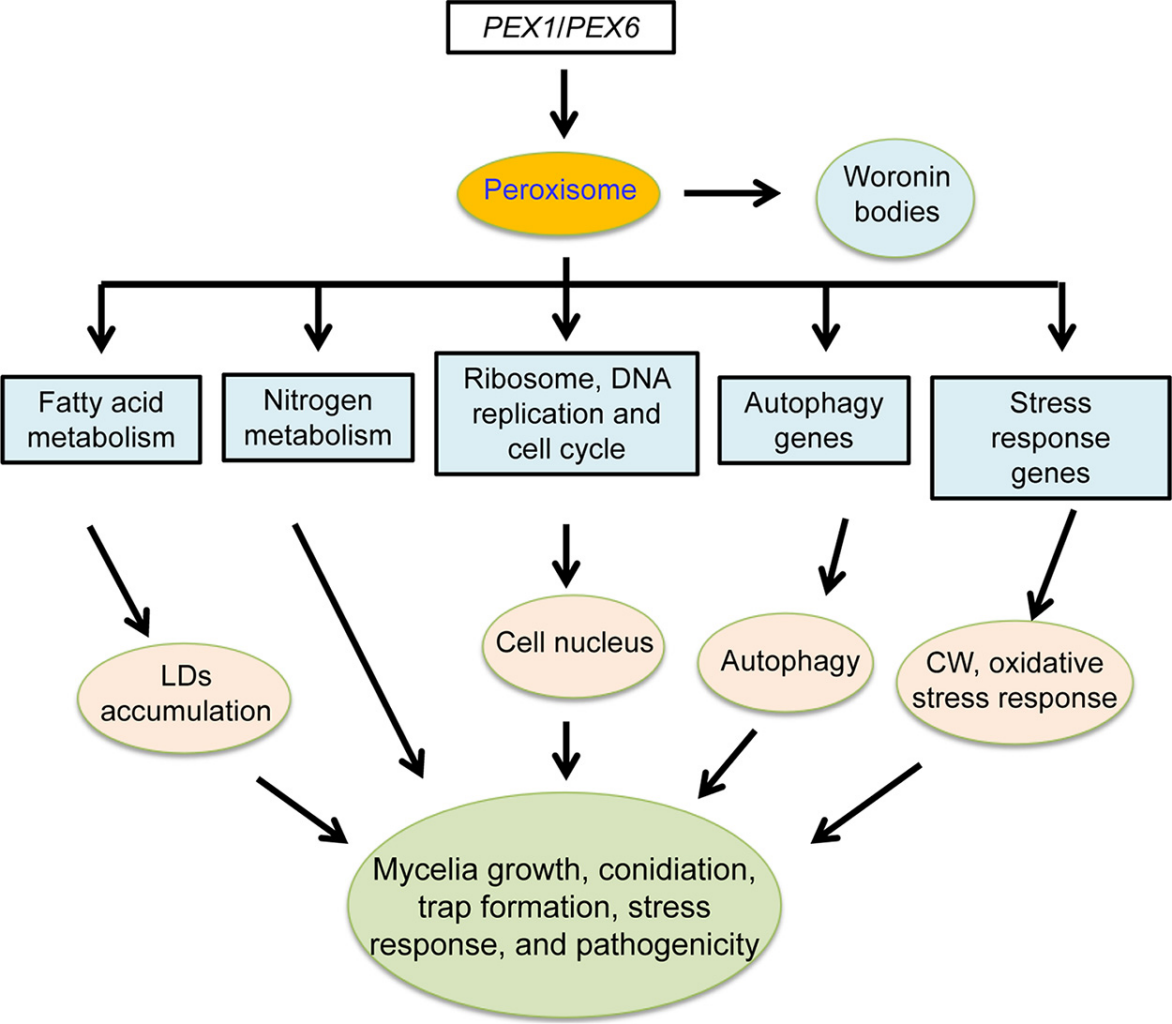
Schematic illustration of the regulation of *AoPEX1 and AoPEX6* in *A. oligospora*. In *A. oligospora*, *AoPEX1* and *AoPEX6* play critical roles in fatty acid metabolism, nutrient metabolism, nucleus formation, autophagy, and oxidative stress processes, thereby regulating hyphal growth, conidiation, and trap formation. CW, cell wall.

By analyzing the phenotypic traits and RT-qPCR and transcriptional data, we propose a putative regulatory pattern of *AoPEX1* and *AoPEX6* in A. oligospora (Fig. 8). *AoPEX1* and *AoPEX6* are indispensable for peroxisome and Woronin body biogenesis. The absence of *AoPEX1* and *AoPEX6* hinders nitrogen and fatty acid metabolism, resulting in reduced mycelial growth and enlarged LDs; disrupts ribosomal function, DNA replication, and the cell cycle, causing a reduction in cell nuclei; alters the expression of genes involved in autophagy, oxidative stress, and cell wall biosynthesis, which contributes to the alteration of related cellular processes. Overall, *AoPEX1* and *AoPEX6* have pleiotropic roles in various cellular processes in A. oligospora, which may have important implications for better understanding the role of *PEX* genes in the growth, development, and pathogenicity of A. oligospora. Furthermore, this data provides a foundation for unraveling the mechanism of lifestyle switching in NT fungi and exploring their potential application in the biocontrol of pathogenic nematodes.

## MATERIALS AND METHODS

### Strains and culture conditions.

The fungus A. oligospora (ATCC24927) and corresponding mutants were stored in the Microbial Library of the Germplasm Bank of Wild Species from Southwest China (Kunming, China). Potato dextrose agar (PDA) medium was prepared for routine culturing of fungal strains at 28°C. Yeast extract peptone dextrose (YPD) medium was used for culturing S. cerevisiae strain FY834 to construct recombinant plasmid vectors (34). Additionally, TG (10 g/L tryptone, 10 g/L glucose, and 20 g/L agar), TYGA (10 g/L tryptone, 5 g/L yeast extract, 10 g/L glucose, 5 g/L molasses, and 20 g/L agar), and CMY [20 g/L maizena (corn starch), 5 g/L yeast extract, and 20 g/L agar] media were used to analyze mycelial growth and related phenotypic traits (47). Escherichia coli strain DH5α (TaKaRa, Shiga, Japan) was used to store the plasmid pCSN44 containing the hygromycin resistance gene (*hph*), and the plasmid PRS426 was used to construct the knockout vector. The nematode Caenorhabditis elegans (N2) was maintained on an oatmeal water medium at 26°C.

### Sequence and phylogenetic analyses of AoPex1 and AoPex6.

Using the orthologs of Pex1 and Pex5 in S. cerevisiae and Aspergillus nidulans as a query, AoPex1 (AOL_s00054g771) and AoPex6 (AOL_s00043g697) were retrieved from A. oligospora. The BLAST algorithm was used to search for Pex1 and Pex6 orthologs in various fungi, and the similarity of the orthologs from different fungi was examined by aligning them using the DNAman software package (version 10.3.307; Lynnon Biosoft, San Ramon, CA, USA) (48) and a neighbor-joining tree was constructed using Mega X software (49).

### Knockout of *AoPEX1* and *AoPEX6* genes.

Taking advantage of the ability of S. cerevisiae FY834 to perform gene self-repair, we used an improved yeast cloning program to construct replacement fragments of *AoPEX1* and *AoPEX6* genes (50, 51). All primers used in this study are listed in Table S4. The upstream and downstream fragments of *AoPEX1* and *AoPEX6* were PCR amplified from A. oligospora using paired primers, and the *hph* gene was amplified using the pSCN44 plasmid as a template (52). The three PCR amplicons and pRS426 plasmid backbone (digested with EcoRI and XhoI) were co-transformed into S. cerevisiae FY834 by electroporation and then inoculated on SC-Ura medium to select recombinant cloned strains (Fig. S2A–B) (34, 52). The constructed vectors (pRS426-Ao*PEX1*-hph and pRS426-Ao*PEX6*-hph) were maintained in E. coli DH5α, and the target fragment for gene disruption was amplified using paired primers and transformed into A. oligospora protoplasts as described previously (47, 53). Transformants were selected on PDAS (PDA supplemented with 0.6 mol/L sucrose) medium containing 200 μg/mL hygromycin (54). The putative transformants were confirmed by PCR amplification and Southern blot analyses (Fig. S2C-D). Southern blotting was performed using the North2South Chemiluminescent Hybridization and Detection Kit (Pierce, Rockford, IL, USA) according to the manufacturer’s instructions.

### Comparison of mycelial growth, morphology, and conidia yield.

The WT and mutant strains were inoculated on PDA, TYGA, and TG media, and their growth rates and colony morphologies were observed after culturing at 28°C. The experiment was repeated thrice for each strain (43, 50). To observe the septum in mycelia, fresh hyphae were stained with 20 μg/mL CFW (Sigma-Aldrich, St. Louis, MO, USA) as described previously (40). The mycelial cell nuclei were visualized by staining with 20 μg/mL DAPI and 20 μg/mL CFW, as previously described (55). The WT and mutant strains cultured on PDA for 7 days were inoculated on PD (200 g/L potatoes, 20 g/L glucose) broth and placed on a shaker at 180 rpm for 3 days. The mycelia were harvested, fixed with 2.5% glutaraldehyde, and observed by TEM. The WT and mutant strains were cultured in CMY medium for 14 days at 28°C. Mycelia were scraped using inoculation loops, and 5 mL of sterile water was added and shaken to obtain the conidia suspension (50 μL). The conidial numbers in the suspensions were counted using a hemocytometer with conidia per cm^2^ of plate culture as an estimate of conidial yield (56).

### Analysis of stress tolerance.

The mycelial plugs of each strain were inoculated onto TG media supplemented with different concentrations of chemical stressors at 28°C for 6 days. The chemical reagents used to determine the stress tolerance of fungal strains were as follows: sorbitol (0.25, 0.5, and 0.75 M) and NaCl (0.1, 0.2, and 0.3 M) as osmotic stressors; sodium dodecyl sulfate (SDS) (0.01, 0.02, and 0.03%) and Congo red (0.05, 0.07, and 0.1 mg/mL) as cell wall stress agents, and menadione (0.01, 0.02, and 0.03 mM) and H_2_O_2_ (2.5, 5, and 10 mM), and oxidative stressors. The diameter of each colony was measured, and the relative growth inhibition (RGI) of each colony was calculated as described previously (47). These experiments were performed at least thrice.

### Trap formation and pathogenicity assays.

WT and mutant strains were incubated on WA (20 g/L agar) plates at 28°C for 3–4 days, and then approximately 300 nematodes (C. elegans) were added to each WA plate to induce trap formation. The number of traps and captured nematodes were observed and quantified using a light microscope (BX51; Olympus, Tokyo, Japan) at specific time points.

### Analysis of LDs and fatty acid utilization.

The WT and mutant strains were cultured on TYGA for 5 days and then stained with 15–20 μL of 10 μg/mL BODIPY staining solution for 30 min. Changes in the size and shape of the lipid droplets were observed using a fluorescence microscope (Leica, Mannheim, Germany). In addition, the WT and mutant strains were treated with MM (2 g/L NaNO_3_, 0.01 g/L FeSO_4_·7H_2_O, 20 g/L glucose, and 20 g/L agar) medium without a carbon source as the basic medium, after adding 50 mM sodium acetate, 0.12% oleic acid, and 0.5% Tween 20 as the only carbon source for culturing for 6 days. The RGI value was calculated by measuring colony diameter as previously described (53, 57).

### Detection of autophagy.

To analyze the changes in the autophagic process in WT and mutant strains, we cultured the strains on TYGA plates with sterile coverslips for 5 days. Subsequently, the stains were treated with 30–50 μL of 100 mg/mL MDC staining solution at 37°C in the dark for 30–40 min, and the images were observed and analyzed by fluorescence microscopy (Leica, Mannheim, Germany).

### RT-qPCR analysis.

Total RNA was extracted from frozen fungal tissues using TRIzol Reagent (Invitrogen, Carlsbad, CA, USA) according to the manufacturer’s protocol. The cDNA was reverse transcribed using a PrimeScript RT reagent kit (TaKaRa, Shiga, Japan) according to the manufacturer’s instructions. RT-qPCR experiments were performed as described previously (42). Relative transcription levels were determined using the 2^- ΔΔCT^ method with the β-tubulin (AOL_s00076g640) gene as the reference (58). All primers used for the RT-qPCR assays are listed in Table S3 in the online supplemental material. Experiments were conducted in triplicate and repeated thrice.

### Transcriptome sequencing and analysis.

The WT and mutant strains were cultured in PDA medium at 28°C for 3 and 5 days, respectively. Three treatment groups with three independent biological replicates were used for each sample. Sequencing of mycelial samples was performed by Shanghai Majorbio Bio-pharm Technology Co., Ltd. (Shanghai, China), and the data were analyzed using the Majorbio Cloud Platform (www.majorbio.com). Subsequently, RT-qPCR was used to verify the transcriptome data (59). The genes and their primers used are listed in Table S3 in the online supplemental material. The transcripts per kilobase million method was used to calculate the expression level of each transcript to identify DEGs (60). Functional enrichment analysis identified the DEGs significantly enriched in the GO terms (*P ≤ *0.05) through GO and KEGG analyses (61).

### Statistical analysis.

One-way analysis of variance (ANOVA) followed by Tukey’s multiple comparison test was used to differentiate the observations, measurements, and estimates. Data are presented as means±standard deviation (SD). GraphPad Prism version 9.00 (GraphPad Software, San Diego, CA, USA) was used for the photographs and statistical analyses from triplicate experiments, and *P < *0.05 was used as the threshold for determining significant differences.

### Data availability.

All data generated or analyzed during this study are included in the published paper and the associated supplemental files. The raw sequence has been deposited to GEO under accession number GSE193953.
