# Supplementary material for: AoPEX1 and AoPEX6 Are Required for Mycelial Growth, Conidiation, Stress Response, Fatty Acid Utilization, and Trap Formation in Arthrobotrys oligospora
Source: Microbiol Spectr. 2022 Mar 24;10(2):e00275-22. doi: 10.1128/spectrum.00275-22 (PMC9045386; doi:10.1128/spectrum.00275-22)
Supplement: SUPPLEMENTAL FILE 1 — Supplemental material. Download SPECTRUM00275-22_Supp_1_seq2.pdf, PDF file, 1.8 MB [file spectrum00275-22_supp_1_seq2.pdf]

SUPPLEMENTAL MATERIALS

Supplementary Figures

Fig. S1. Neighbor-joining phylogenetic tree based on the amino acid sequence of Pex1 and Pex6 homologous proteins from different fungi. GenBank accession numbers are provided in brackets. Numbers below nodes indicate the bootstrap value. The bar marker indicates the genetic distance, which is proportional to the number.

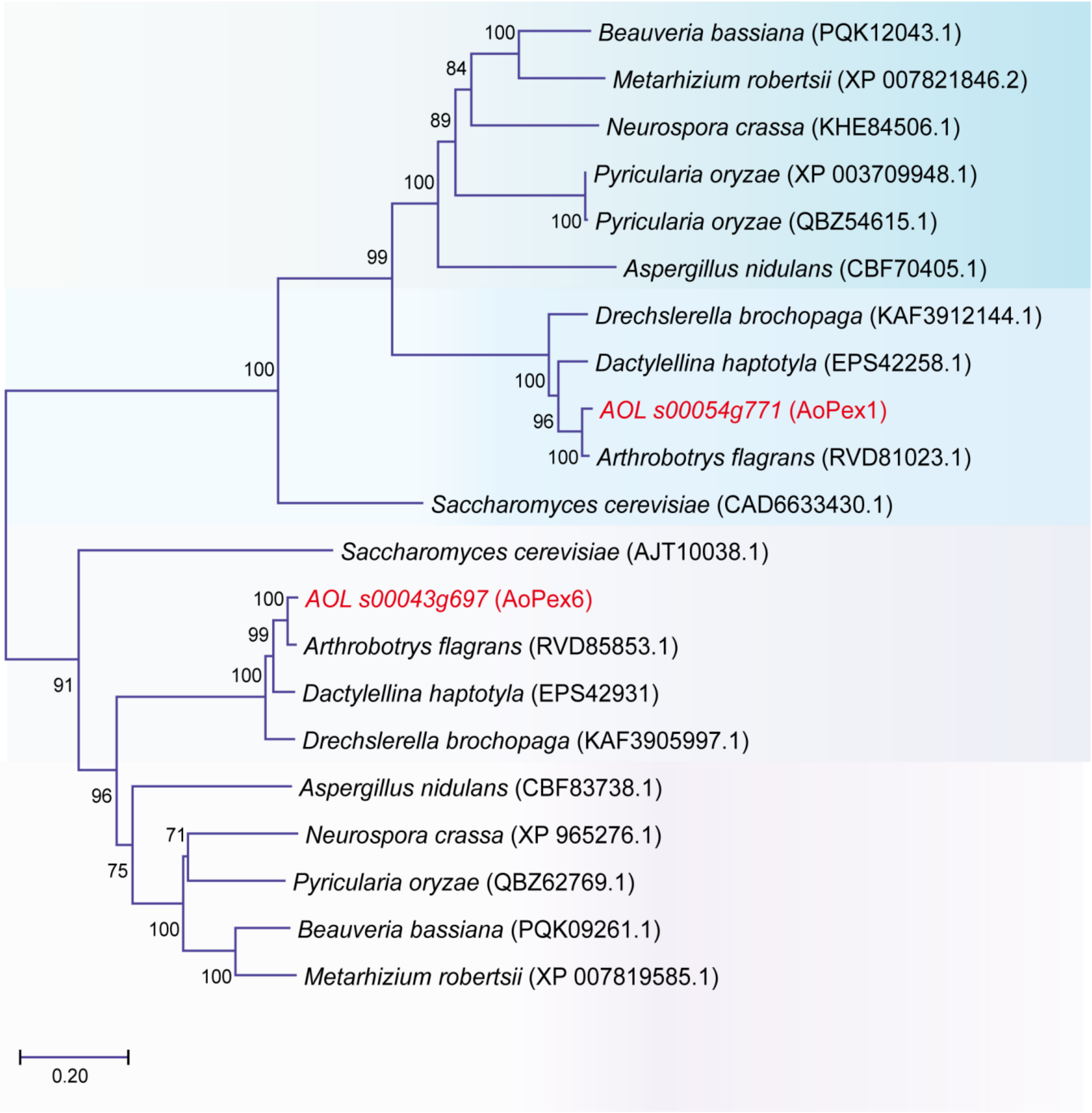

Fig. S2. *AoPEX1* and *AoPEX6* knock-out and verification in *A. oligospora*. Diagrammatic sketch of homologous recombination. (B) The diagrammatic sketch of homologous recombination of *AoPEX1* and *AoPEX6* and the homologous flanks of the target gene. (C) *AoPEX1* and *AoPEX6* deletion transformants (Δ*Aopex1* and Δ*Aopex6*) were confirmed by PCR method. (D) Southern blotting analysis of wild-type (WT) and transformants.

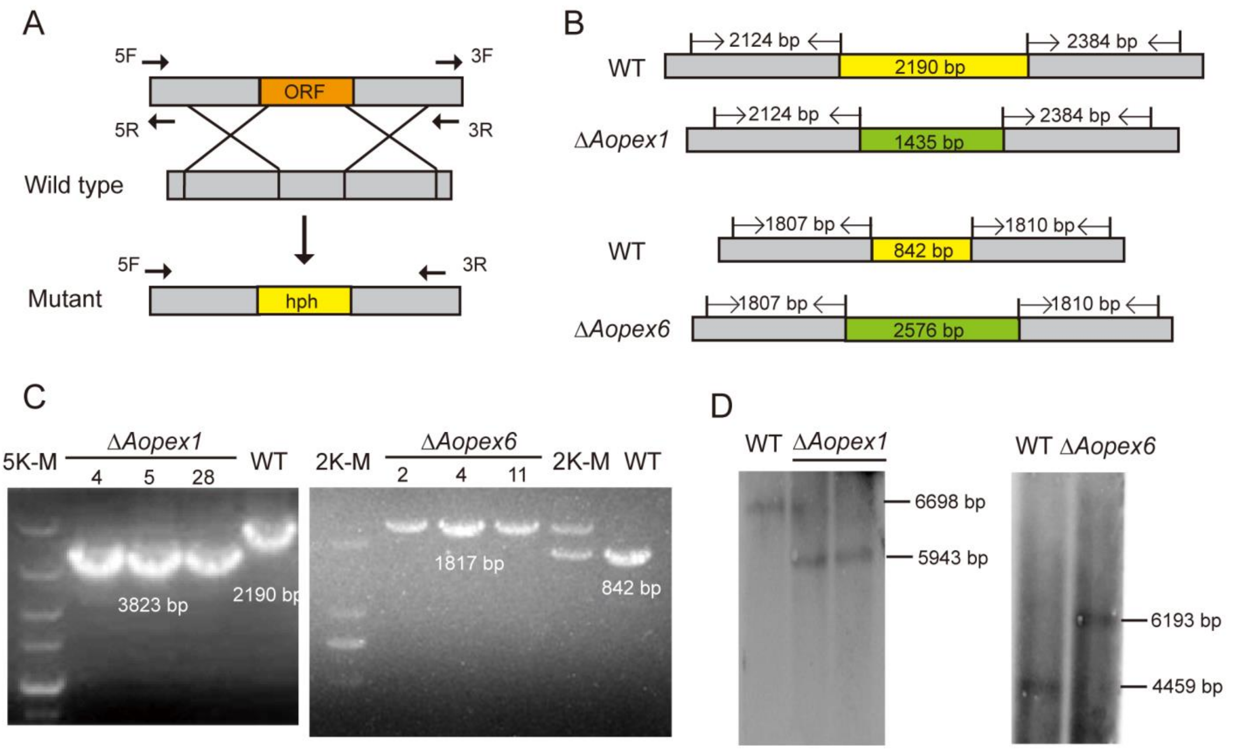

Fig. S3. Differentially expressed genes (DEGs) associated with sporulation.

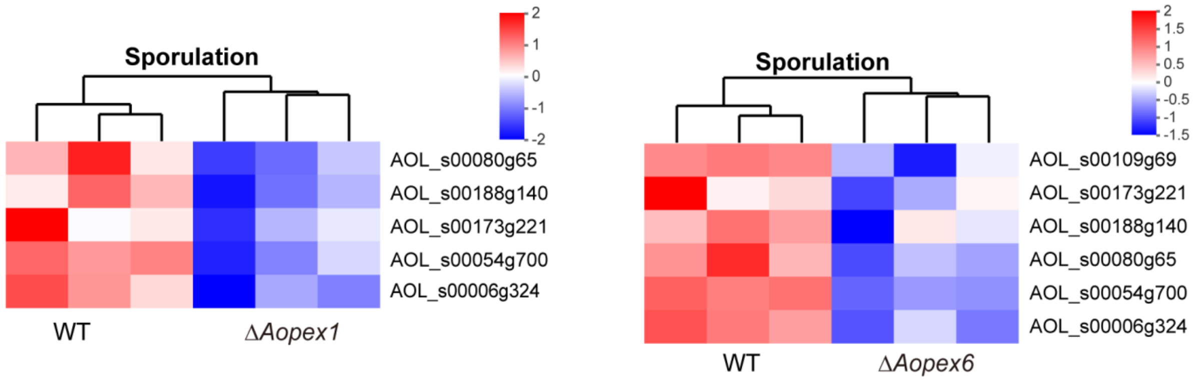

Fig. S4. Comparison of stress tolerance to osmotic agents between wild-type (WT) and mutant strains of *A. oligospora*. (A) Colony morphologies of the WT and mutant strains incubated on TG medium supplemented with sorbitol or NaCl. (B) Relative growth inhibition (RGI) values of the WT and mutants after incubation on TG medium supplemented with 0.25-0.75 M sorbitol for 6 days. (C) RGI values for the WT and mutant strains after incubation on TG medium supplemented with 0.10-0.30 M NaCl for 6 days. The asterisk (B and C) indicates a significant difference between the mutant and the WT strains (*$p < 0.05$, **$p < 0.01$, ***$p < 0.001$).

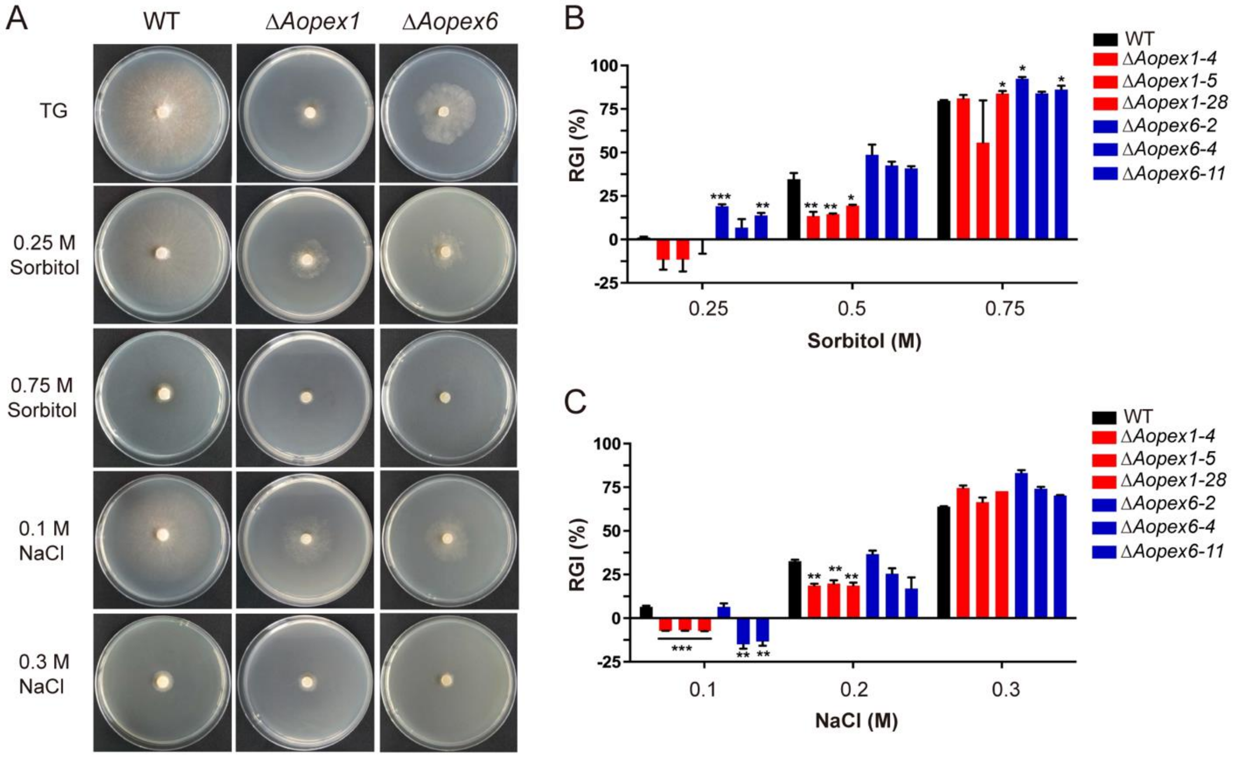

Fig. S5. Comparison of stress tolerance to cell wall-perturbing agents between wild-type (WT) and mutant strains of *A. oligospora*. (A) Colony morphologies of the WT and mutant strains incubated on TG medium supplemented with SDS or Congo red. (B) Relative growth inhibition (RGI) values of the WT and mutants after incubation on TG medium supplemented with 0.01- 0.03% SDS for 6 days. (C) RGI values for the WT and mutant strains after incubation on TG medium supplemented with 0.05- 0.1 mg/mL Congo red for 6 days. The asterisk (B and C) indicates a significant difference between the mutant and the WT strains (**p* < 0.05, ***p* < 0.01, ****p* < 0.001).

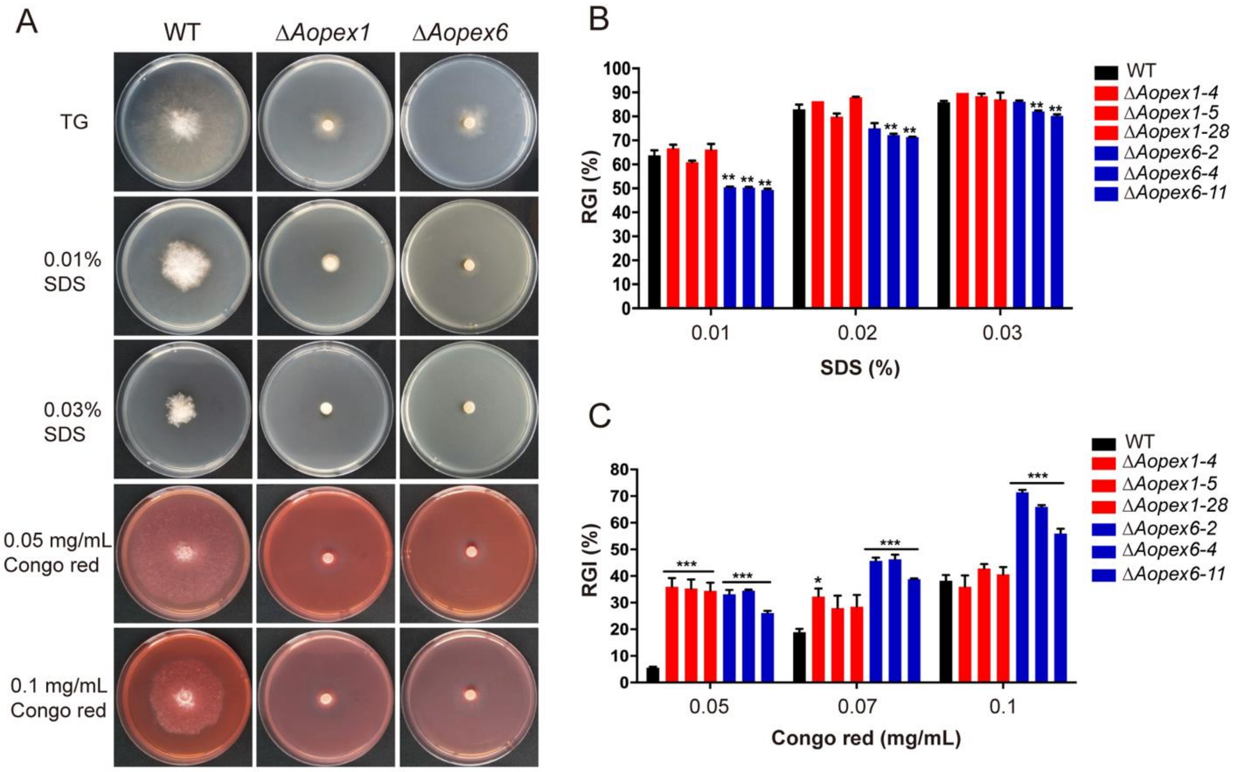

Fig. S6. Principal component analysis. Different shapes and colors represent the WT strains, Δ*Aopex1*, and Δ*Aopex6* mutant strains at 3 d and 5 d.

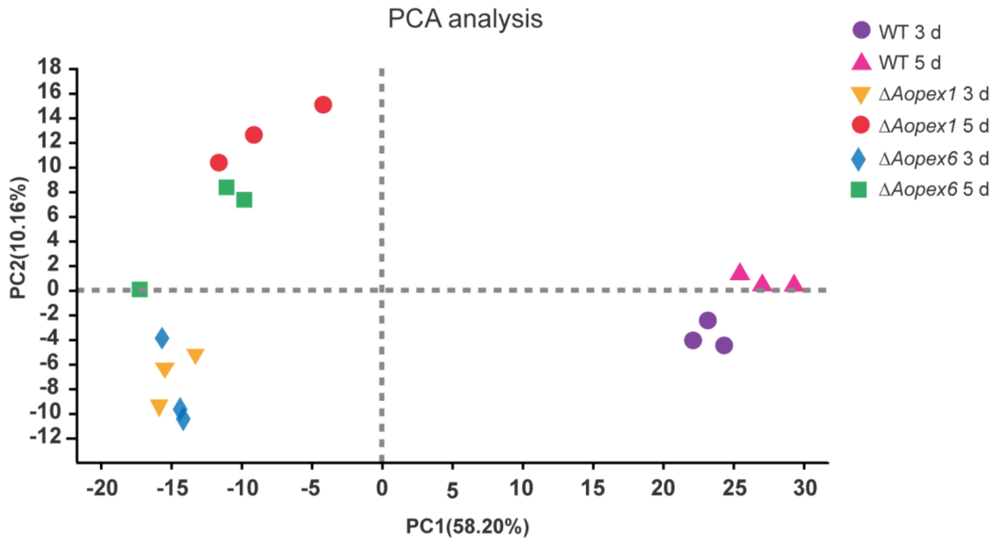

Fig. S7. Reverse transcription qualitative PCR (RT-qPCR) was used to verify the transcriptome data between the wild-type (WT), Δ*Aopex1*, and Δ*Aopex6* mutant strains at 3 d and 5 d.

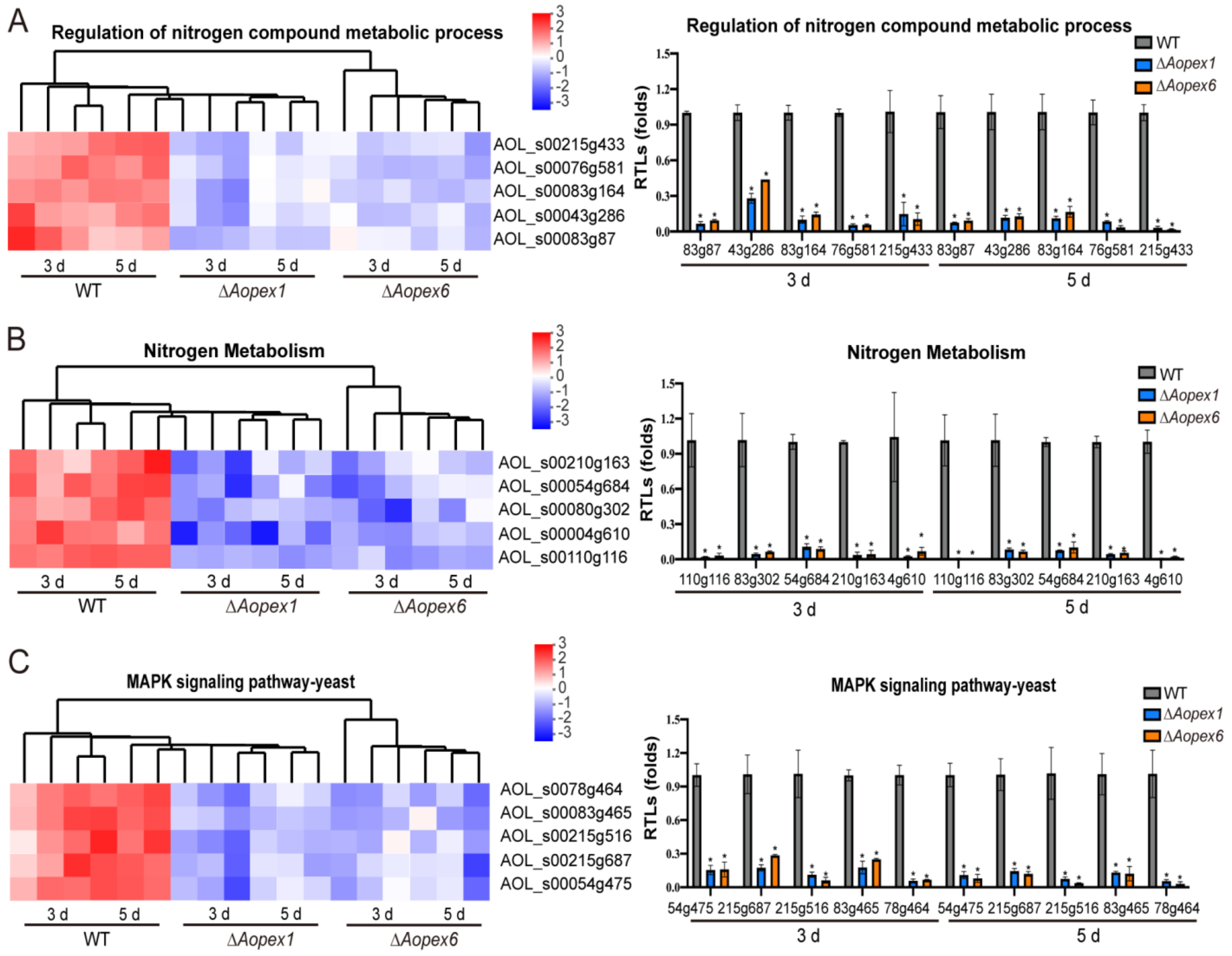

Fig. S8. UpSet plot for DEGs in the Δ*Aopex1* and Δ*Aopex6* mutant versus WT strain. The bar chart at the bottom left represents the number of DEGs in the Δ*Aopex1* and Δ*Aopex6* mutant strains versus the WT strain at 3 d and 5 d. The dotted line at the bottom right shows the number of DEGs in the different groups. Purple indicates the number of DEGs shared by four groups, a single dot indicates the unique DEGs of each group, and the dotted line indicates the intersection of different groups.

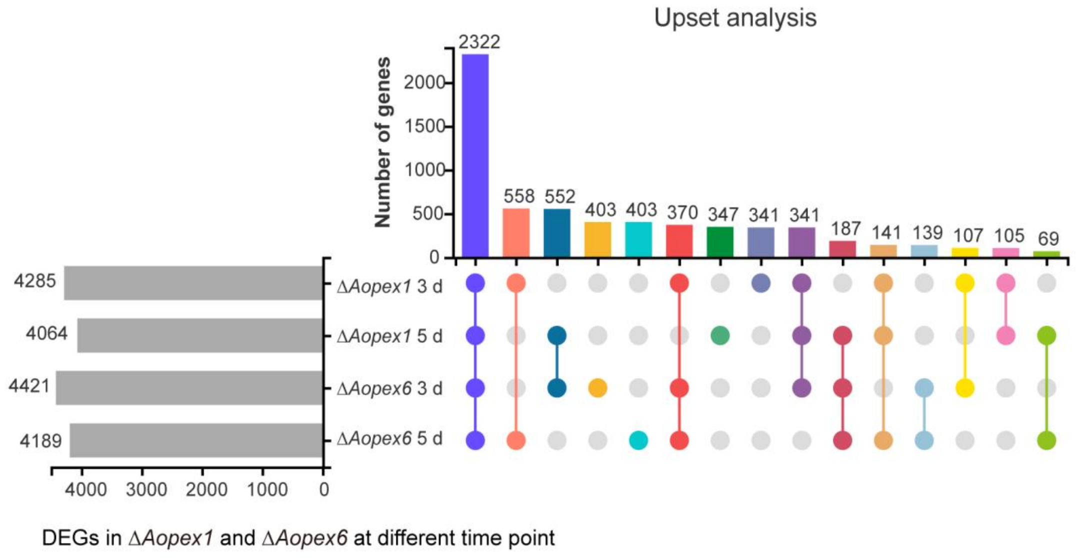

Fig. S9. Gene Ontology (GO) enrichment of differentially expressed genes (DEGs) between the wild-type (WT) strain, Δ*Aopex1*, and Δ*Aopex6* mutant strains at 3 d and 5 d. (A) GO enrichment analysis of DEGs in the Δ*Aopex1* mutant versus WT strain at 3 d and 5 d. (B) GO enrichment analysis of DEGs in the Δ*Aopex6* mutant versus WT strain at 3 d and 5 d. The red and green columns represent significant upregulation and downregulation, respectively.

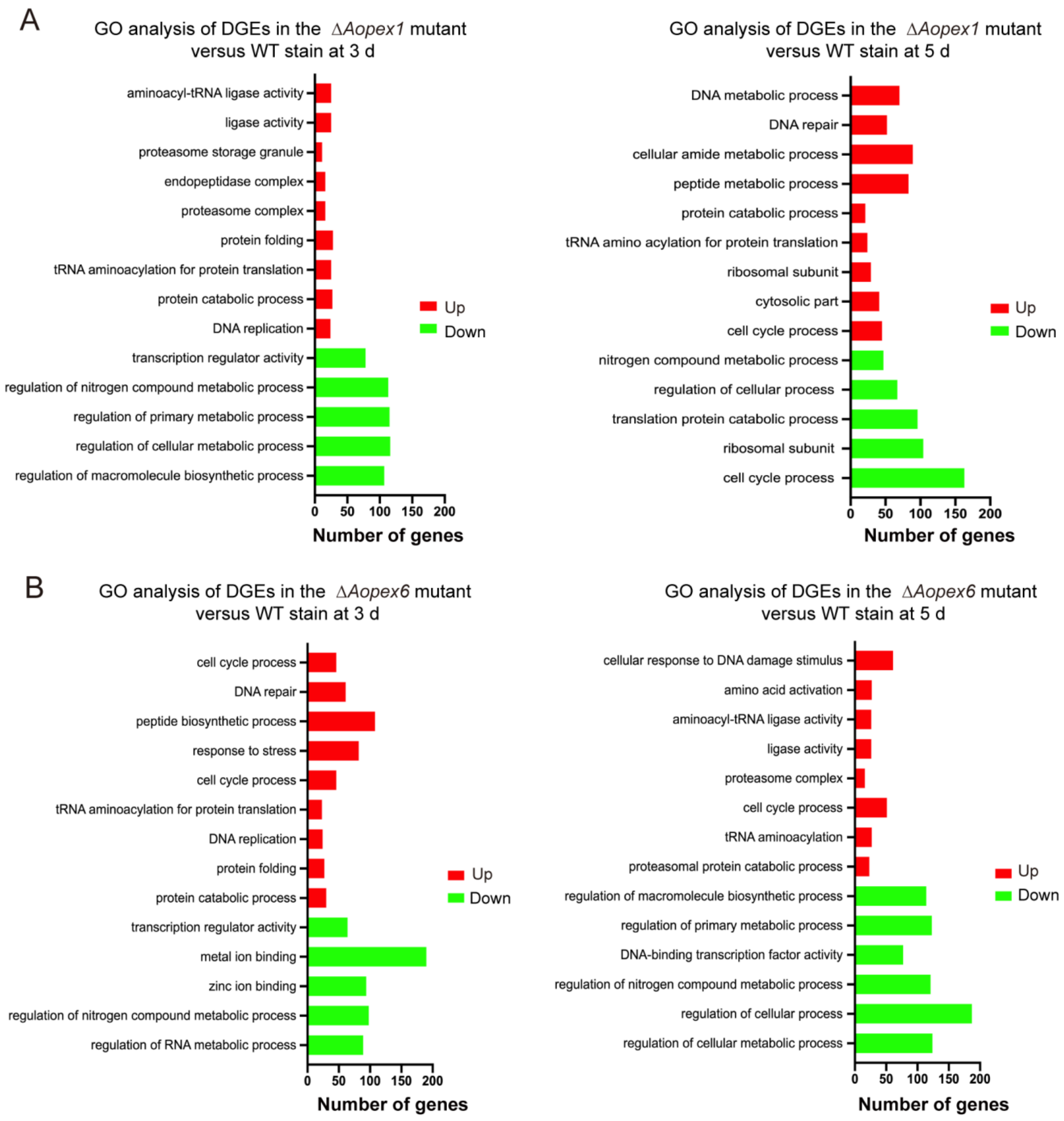

Fig. S10. Differentially expressed genes (DEGs) associated with autophagy.

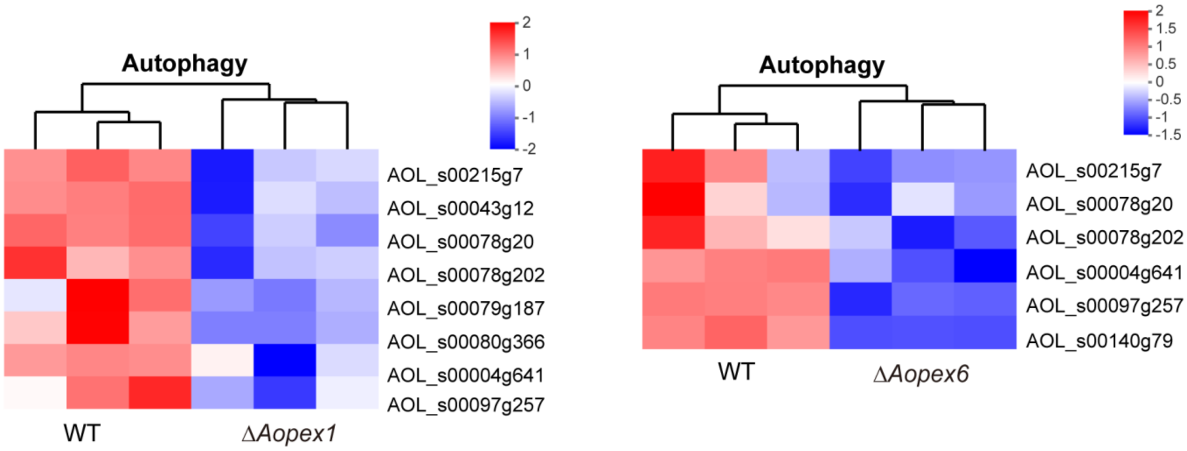

Supplementary tables

Table S1. Statistics of reads and mapping rate for the wild-type (WT) strain (A), Δ*Aopex1* (F), and Δ*Aopex6* (E) mutant strains at different time points.

Sample	Total reads	Total mapped	Multiple mapped	Uniquely mapped
A_3d_1	47960048	46024268(95.96%)	176266(0.37%)	45848002(95.6%)
A_3d_2	53301834	51076298(95.82%)	201610(0.38%)	50874688(95.45%)
A_3d_3	47679106	45640603(95.72%)	219181(0.46%)	45421422(95.26%)
A_5d_1	50743682	48597220(95.77%)	171976(0.34%)	48425244(95.43%)
A_5d_2	49912364	47866869(95.9%)	170642(0.34%)	47696227(95.56%)
A_5d_3	49596520	47834250(96.45%)	187232(0.38%)	47647018(96.07%)
E_3d_1	45701444	43890980(96.04%)	175466(0.38%)	43715514(95.65%)
E_3d_2	43837774	41868926(95.51%)	169011(0.39%)	41699915(95.12%)
E_3d_3	45264678	43496993(96.09%)	167863(0.37%)	43329130(95.72%)
E_5d_1	50843760	48754776(95.89%)	196944(0.39%)	48557832(95.5%)
E_5d_2	42585932	40799797(95.81%)	145294(0.34%)	40654503(95.46%)
E_5d_3	52431284	50357586(96.04%)	199352(0.38%)	50158234(95.66%)
F_3d_1	44362998	42615171(96.06%)	183696(0.41%)	42431475(95.65%)
F_3d_2	41252746	39600804(96.0%)	188814(0.46%)	39411990(95.54%)

F_3d_3	44242996	42408182(95.85%)	222612(0.5%)	42185570(95.35%)
F_5d_1	41619532	39879193(95.82%)	141544(0.34%)	39737649(95.48%)
F_5d_2	44738554	42864293(95.81%)	153313(0.34%)	42710980(95.47%)
F_5d_3	50972364	48702637(95.55%)	161721(0.32%)	48540916(95.23%)

Table S2. Statistics of reads, Phred-like quality scores and GC content for the wild-type (WT) strain (A), Δ*Aopex1* (F), and Δ*Aopex 6* (E) mutant at different time points.

Sample	Raw reads	Raw bases	Clean reads	Clean bases	Error rate(%)	Q20(%)	Q30(%)	GC content(%)
A_3d_1	48307866	7294487766	47960048	7053941103	0.0262	97.5	93.02	48.1
A_3d_2	53720066	8111729966	53301834	7834367820	0.0264	97.42	92.85	47.87
A_3d_3	48037264	7253626864	47679106	7029038824	0.0267	97.32	92.6	47.67
A_5d_1	51081842	7713358142	50743682	7488905945	0.0264	97.42	92.84	48
A_5d_2	50346484	7602319084	49912364	7343481951	0.0266	97.35	92.69	48.03
A_5d_3	49880518	7531958218	49596520	7299728862	0.025	98.07	94.02	47.89
E_3d_1	46088698	6959393398	45701444	6772960726	0.0266	97.37	92.71	48.1
E_3d_2	44222420	6677585420	43837774	6478216752	0.0266	97.37	92.73	48.4
E_3d_3	45748954	6908092054	45264678	6693151697	0.0266	97.33	92.65	48.19
E_5d_1	51289210	7744670710	50843760	7497681132	0.0265	97.4	92.8	47.88
E_5d_2	42999608	6492940808	42585932	6296089372	0.0266	97.36	92.73	47.41
E_5d_3	52827488	7976950688	52431284	7736618613	0.0264	97.45	92.9	48.1
F_3d_1	44818670	6767619170	44362998	6557569636	0.0266	97.34	92.67	47.93
F_3d_2	41631518	6286359218	41252746	6114751203	0.0265	97.38	92.77	48
F_3d_3	44703994	6750303094	44242996	6555639976	0.0267	97.29	92.57	47.85
F_5d_1	41969332	6337369132	41619532	6176903380	0.0268	97.28	92.51	47.75
F_5d_2	45049418	6802462118	44738554	6643591240	0.0266	97.36	92.69	47.84
F_5d_3	51390266	7759930166	50972364	7474451621	0.0263	97.45	92.93	47.9

Table S3. Paired primers used for qRT-PCR in *A. oligospora*.

Primers	Sequence (5′-3′)	Sequence (3′-5′)
AOL_s00083g87	5F-CGACACAACATGCTCTTGTAAA	3R-GAGATTAACAATGTCAGCGCTT
AOL_s00043g286	5F-GATTTTGAAAAGTTGCAGGCAG	3R-GAAATCCGTGGTGTGCAATTAT
AOL_s00083g164	5F-TTTTACTGACTGCTTCAACTGC	3R-GAATATCAGTTGGACGCATGAC
AOL_s00076g581	5F-ATCACCGTCGATATACAACGAA	3R-ATTATCTCGAGATTTCCCGCAT
AOL_s00215g433	5F-GTATGTGTATGCTCCGAGAAGA	3R-AAAGTAATAAACCTGCATGGCG
AOL_s00110g116	5F-ACGATTCTGCCAATCTTTCATG	3R-CCTTCCCAGATGTTCTTGTAGT

AOL_s00080g302	5F-GTACCCTATCCGAAAACCTCTC	3R-GGTAGTTCTGAGAGTGCCTTAA
AOL_s00054g684	5F-GATGTCTGGCTGAAGAACATTC	3R-CATCATAGACGAAACCGTGAAC
AOL_s00210g163	5F-AGAAGTGTGAAATGGCTGAAAC	3R-GCGAGTTCTGTAAGTTTTTGGT
AOL_s00004g610	5F-AGTCGCGAACTCAAATGTATTG	3R-TCGAATAGCCATAAGTCCAGTG
AOL_s00054g475	5F-GGCATGGCTATTCGAATTCTAC	3R-GATTTGGACTAGTTGGCGATTC
AOL_s00215g687	5F-CACGTTTCAGTACTACGCTTTC	3R-GAGCTGCATAAGAAGATTGTGG
AOL_s00215g516	5F-GCAAAGAGTCTACAAGTGTTGG	3R-CTTTTTCTTCGACTGGATAGCG
AOL_s00083g465	5F-TCAAGACACCCATTGTTACTCA	3R-TTTCGTCGAACAATGAGGAAAC
AOL_s00078g464	5F-GGTGTATTTGTTGGTGTAGCAG	3R-TTATTACTGGTGCCTTGTCCTG
AOL_s00076g640 (*tub*)	5F-CCACCTTCGTCGGTAACTC	3R-TCGTCCATACCCTCACCAG

Table S4. List of primers for gene disruption in this study.

**Primers**	**Sequence (5′-3′)**	**Description**
AoPEX1-5F	GTAACGCCAGGGTTTTCCCAGTCACGACGCGACGGGTAGAACAGTGAGA	Amplify the *AoPEX1* gene 5’ flank
AoPEX1-5R	ATCCACTTAACGTTACTGAAATCTCCAACTTGTATGGGTTGTGGGTGG	
AoPEX1-3F	CTCCTTCAATATCATCTTCTGTCTCCGACACTCGCTATGCCCCAATCT	Amplify the *AoPEX1* gene 3’ flank
AoPEX1-3R	GCGGATAACAATTTCACACAGGAAACAGCAAGCTGTACGGGTCTAAGGAA	
AoPEX6-5F	GTAACGCCAGGGTTTTCCCAGTCACGACGAGAGGAGGATGACGGAGGTT	Amplify the *AoPEX6* gene 5’ flank
AoPEX6-5R	ATCCACTTAACGTTACTGAAATCTCCAACATTGGAGGTGGGTTGTTCG	
AoPEX6-3F	CTCCTTCAATATCATCTTCTGTCTCCGACTCAAGAGTGCGGTTATGGAA	Amplify the *AoPEX6* gene 3’ flank
AoPEX6-3R	GCGGATAACAATTTCACACAGGAAACAGCTGGTTGGGACGTGCTGTAT	
Hph-F	GTCGGAGACAGAAGATGATATTGAAGGAGC	Amplify the *hph* cassette
Hph-R	GTTGGAGATTTCAGTAACGTTAAGTGGAT	
AoPEX1-YZR	GGAGGCGAGCAGAGTTTT	
AoPEX6-YZF	CAAATCCCTCCTACTGCC	
AoPEX6-YZR	TTCACTCATACCGTCCAA	
AoPEX1-TZF	CAATCTTCGCCAACTGTCC	Make Southern blotting probe
AoPEX1-TZR	AATGTCTTCTAGTCCGATGCTC	

AoPEX6-TZF	GCCAGAGTTATTCGCAAAGG
AoPEX6-TZR	GGAGACACCCAAGAACAACAT
